# Comparative Genomics Reveals Thermal Adaptation and a High Metabolic Diversity in “*Candidatus* Bathyarchaeia”

**DOI:** 10.1128/mSystems.00252-21

**Published:** 2021-07-20

**Authors:** Yan-Ling Qi, Paul N. Evans, Yu-Xian Li, Yang-Zhi Rao, Yan-Ni Qu, Sha Tan, Jian-Yu Jiao, Ya-Ting Chen, Brian P. Hedlund, Wen-Sheng Shu, Zheng-Shuang Hua, Wen-Jun Li

**Affiliations:** a State Key Laboratory of Biocontrol, Guangdong Provincial Key Laboratory of Plant Resources and Southern Marine Science and Engineering Guangdong Laboratory (Zhuhai), School of Life Sciences, Sun Yat-Sen University, Guangzhou, People’s Republic of China; b The Australian Centre for Ecogenomics, School of Chemistry and Molecular Biosciences, University of Queensland, St Lucia, Queensland, Australia; c School of Life Sciences, University of Nevada Las Vegas, Las Vegas, Nevada, USA; d Nevada Institute of Personalized Medicine, University of Nevada Las Vegas, Las Vegas, Nevada, USA; e School of Life Sciences, South China Normal University, Guangzhou, People’s Republic of China; f Department of Environmental Science and Engineering, University of Science and Technology of China, Hefei, People’s Republic of China; g State Key Laboratory of Desert and Oasis Ecology, Xinjiang Institute of Ecology and Geography, Chinese Academy of Sciences, Urumqi, People’s Republic of China; Purdue University

**Keywords:** *Ca*. Bathyarchaeia, polysaccharide degradation, alkane metabolism, thermal adaptation, horizontal gene transfer

## Abstract

“*Candidatus* Bathyarchaeia” is a phylogenetically diverse and widely distributed lineage often in high abundance in anoxic submarine sediments; however, their evolution and ecological roles in terrestrial geothermal habitats are poorly understood. In the present study, 35 *Ca*. Bathyarchaeia metagenome-assembled genomes (MAGs) were recovered from hot spring sediments in Tibet and Yunnan, China. Phylogenetic analysis revealed all MAGs of *Ca*. Bathyarchaeia can be classified into 7 orders and 15 families. Among them, 4 families have been first discovered in the present study, significantly expanding the known diversity of *Ca*. Bathyarchaeia. Comparative genomics demonstrated *Ca*. Bathyarchaeia MAGs from thermal habitats to encode a large variety of genes related to carbohydrate degradation, which are likely a metabolic adaptation of these organisms to a lifestyle at high temperatures. At least two families are potential methanogens/alkanotrophs, indicating a potential for the catalysis of short-chain hydrocarbons. Three MAGs from Family-7.3 are identified as alkanotrophs due to the detection of an Mcr complex. Family-2 contains the largest number of genes relevant to alkyl-CoM transformation, indicating the potential for methylotrophic methanogenesis, although their evolutionary history suggests the ancestor of *Ca*. Bathyarchaeia was unable to metabolize alkanes. Subsequent lineages have acquired the ability via horizontal gene transfer. Overall, our study significantly expands our knowledge and understanding of the metabolic capabilities, habitat adaptations, and evolution of *Ca*. Bathyarchaeia in thermal environments.

**IMPORTANCE** *Ca*. Bathyarchaeia MAGs from terrestrial hot spring habitats are poorly revealed, though they have been studied extensively in marine ecosystems. In this study, we uncovered the metabolic capabilities and ecological role of *Ca*. Bathyarchaeia in hot springs and give a comprehensive comparative analysis between thermal and nonthermal habitats to reveal the thermal adaptability of *Ca*. Bathyarchaeia. Also, we attempt to determine the evolutionary history of methane/alkane metabolism in *Ca*. Bathyarchaeia, since it appears to be the first archaea beyond *Euryarchaeota* which contains the *mcrABG* genes. The reclassification of *Ca*. Bathyarchaeia and significant genomic differences among different lineages largely expand our knowledge on these cosmopolitan archaea, which will be beneficial in guiding the future studies.

## INTRODUCTION

*Candidatus* Bathyarchaeia were originally named Miscellaneous Crenarchaeotal Group (MCG) and first discovered in hot springs (1) and in coastal subseafloor sediments (2) based on community 16S rRNA gene sequencing projects. In subsequent single-cell genomic and metagenomic surveys, the group was designated as a new archaeal phylum and named *Ca*. Bathyarchaeota (3, 4), which represents a phylogenetically diverse group that is ubiquitously distributed among a wide variety of ecosystems, such as hypersaline (5, 6), marine (3, 7), and freshwater (8) sediments, with particularly high abundance (up to 100% of total archaeal abundance) in marine sediments (3, 9–12). More recently, according to the Genome Taxonomy Database (GTDB), this lineage may alternatively be considered a class, *Ca*. Bathyarchaeia, belonging to the phylum *Thermoproteota* (13). Since first being named, the current *Ca*. Bathyarchaeia group has been divided into up to 23 subgroups on the basis of 16S rRNA gene phylogenies (12, 14–16). Their ubiquity and frequent predominance in natural anaerobic microbial communities is likely due to their capacity to metabolize multiple types of organic substrates, such as detrital proteins, aromatic compounds, lignin, and extracellular carbohydrates (3, 4, 17–22). Also, a recent study revealed genes for the methyl-coenzyme M reductase (*mcr*) complex in this lineage, expanding its distribution beyond traditional methanogenic/methanotrophic archaeal lineages (23). Nevertheless, it has since been deduced that the *mcr* genes in *Ca*. Bathyarchaeia likely perform alkane oxidation rather than methane metabolism (24–27). It remains unclear whether this capacity has been acquired via horizontal gene transfer (HGT) or was inherited vertically. Also, knowledge gaps regarding the evolutionary history of other genes relevant to methane/alkane metabolism persist, obscuring the possible role of the Mcr complex in *Ca*. Bathyarchaeia.

Here, using genome-resolved metagenomics, we constructed 35 *Ca*. Bathyarchaeia metagenome-assembled genomes (MAGs) from the hot spring sediments of Yunnan and Tibet in China and aim to address the following: (i) their taxonomic status by using the software GTDB (28) to reclassify the phylogeny at the order and family levels; (ii) their metabolic characteristics in hot spring habitats; (iii) functional differences between *Ca*. Bathyarchaeia from thermal and nonthermal environments, with 60 additional *Ca*. Bathyarchaeia MAGs from public databases to show adaptations to thermal environments; and (iv) the evolutionary history and origin of the potential methane or alkane metabolism of specific lineages.

## RESULTS AND DISCUSSION

### General genomic features, phylogenetic placement, and distribution of *Ca*. Bathyarchaeia.

A total of 35 *Ca*. Bathyarchaeia MAGs were successfully reconstructed from 12 metagenomic sequence data sets that were generated from sediments of hot springs in Tibet and Tengchong, China (Table 1; see also Table S1 and Fig. S1a in the supplemental material), which span a wide range of temperatures from 56.9 to 83.0°C and pH values ranging from 6.0 to 7.6. Large variations in the relative abundance of these *Ca*. Bathyarchaeia (from 0.05% to 10%) were observed in the 12 metagenomic data sets from these sediments (Fig. S1b). A total of 19 and 16 MAGs were obtained from the Tengchong and Tibet hot spring metagenomes, respectively (Fig. S1a). The genomic sizes of these MAGs are approximately 1.08 to 1.98 Mbp (average 1.48 Mbp) with an average of 1,640 genes encoded (Table S1). Of these 35 genomes, 17 are considered “high” and 18 considered “medium” quality, ranging from 62.6 to 99.1% completeness, with nearly undetectable contamination at an average level of 1.13% according to Bowers et al. (29) (Table S1). Along with the MAGs from hot spring ecosystems generated in this study, 60 MAGs with medium and high quality (completeness of ≥50.34%) available in the NCBI and IMG databases were coinvestigated. These 60 MAGs are widely distributed among many habitat types, including bioreactor, estuary (18, 21), freshwater (23, 30–32), hydrothermal vent (17, 22, 33, 34), ocean (32, 35–37), and soil (32, 37) environments (Data Set S1). The wide distribution and broad phylogenetic diversity of these *Ca*. Bathyarchaeia MAGs suggest a high level of genomic and/or phenotypic plasticity, which may allow them to occupy a wide range of habitats.

**TABLE 1:** General genomic features of the *Ca*. Bathyarchaeia MAGs reconstructed from hot spring sediments in present study

Family	Family-1.1	Family-1.2	Family-1.3	Family-3.1	Family-4.1	Family-4.3	Family-5	Family-7.2	Family-7.4
No. of genomes	2	1	3	1	3	4	4	10	7
No. of scaffolds	143–181	14	74–194	30	12–32	42–187	43–162	35–262	9–358
Genome size (Mb)	1.11–1.67	1.41	1.30–1.98	1.39	1.33–1.75	1.31–1.70	1.32–1.90	1.1–1.74	1.08–1.71
GC content (%)	46.7–47.3	41.0	40.6–41.4	42.8	30.9–52.8	45.4–51.8	43.2–52.1	41.2–45.9	41.6–45.9
No. of protein coding genes	1,184–1,724	1,442	1,526–2,113	1,496	1,439–1,826	1,610–1,991	1,517–2,117	1,197–1,907	1,331–1,929
Coding density (%)	76.8–79.9	91.0	85.0–86.8	87.7	84.6–88.8	83.9–87.7	84.1–87.2	86.4–91.1	86.0–92.6
No. of genes annotated by KO	658 (46%)	765 (53%)	777 (41%)	773 (52%)	798 (50%)	799 (44%)	818 (45%)	738 (49%)	753 (47%)
No. of genes annotated by COG	985 (68%)	1,085 (75%)	1,248 (65%)	1,105 (74%)	1,186 (74%)	1,204 (66%)	1,244 (69%)	1,105 (73%)	1,092 (69%)
No. of genes annotated by arCOG	996 (69%)	1,095 (76%)	1,257 (66%)	1,133 (76%)	1,227 (76%)	1,242 (68%)	1,259 (70%)	1,117 (74%)	1,118 (70%)
No. of genes annotated by pfam	1,012 (70%)	1,130 (78%)	1,311 (69%)	1,170 (78%)	1,245 (77%)	1,273 (70%)	1,308 (72%)	1,147 (76%)	1,135 (71%)
Completeness^*a*^	68.6–85.6	93.9	74.7–98.0	98.1	96.3–98.1	69.5–97.2	62.6–94.9	74.5–99.1	75.4–99.1
Contamination^*a*^	1.25–1.94	0.93	0–2.43	0.47	0–0.93	0–0.97	0–0.93	0–4.67	0–2.80

a Completeness and contamination were estimated by CheckM (Parks et al. [65]).

10.1128/mSystems.00252-21.1 FIG S1 Sampling sites in this study. (a) Sampling sites collected from Tibet and Yunnan, China. Three of the 11 are from Tengchong in Yunnan and eight are from Tibet. The numbers in brackets represent the number of *Ca*. Bathyarchaeia MAGs reconstructed from the corresponding sampling site. (b) Relative abundances of *Ca*. Bathyarchaeia in the corresponding microbial communities. Download FIG S1, PDF file, 1.0 MB. Copyright © 2021 Qi et al. 2021 Qi et al. https://creativecommons.org/licenses/by/4.0/ This content is distributed under the terms of the Creative Commons Attribution 4.0 International license.

10.1128/mSystems.00252-21.6 TABLE S1 Overview of 35 *Ca*. Bathyarchaeia MAGs from hot springs. Download Table S1, PDF file, 0.3 MB. Copyright © 2021 Qi et al. 2021 Qi et al. https://creativecommons.org/licenses/by/4.0/ This content is distributed under the terms of the Creative Commons Attribution 4.0 International license.

10.1128/mSystems.00252-21.7 DATA SET S1 The genome characteristics of 60 *Ca*. Bathyarchaeia draft genomes downloaded from public databases in this research. Download Data Set S1, XLS file, 0.05 MB. Copyright © 2021 Qi et al. 2021 Qi et al. https://creativecommons.org/licenses/by/4.0/ This content is distributed under the terms of the Creative Commons Attribution 4.0 International license.

**FIG 1:**
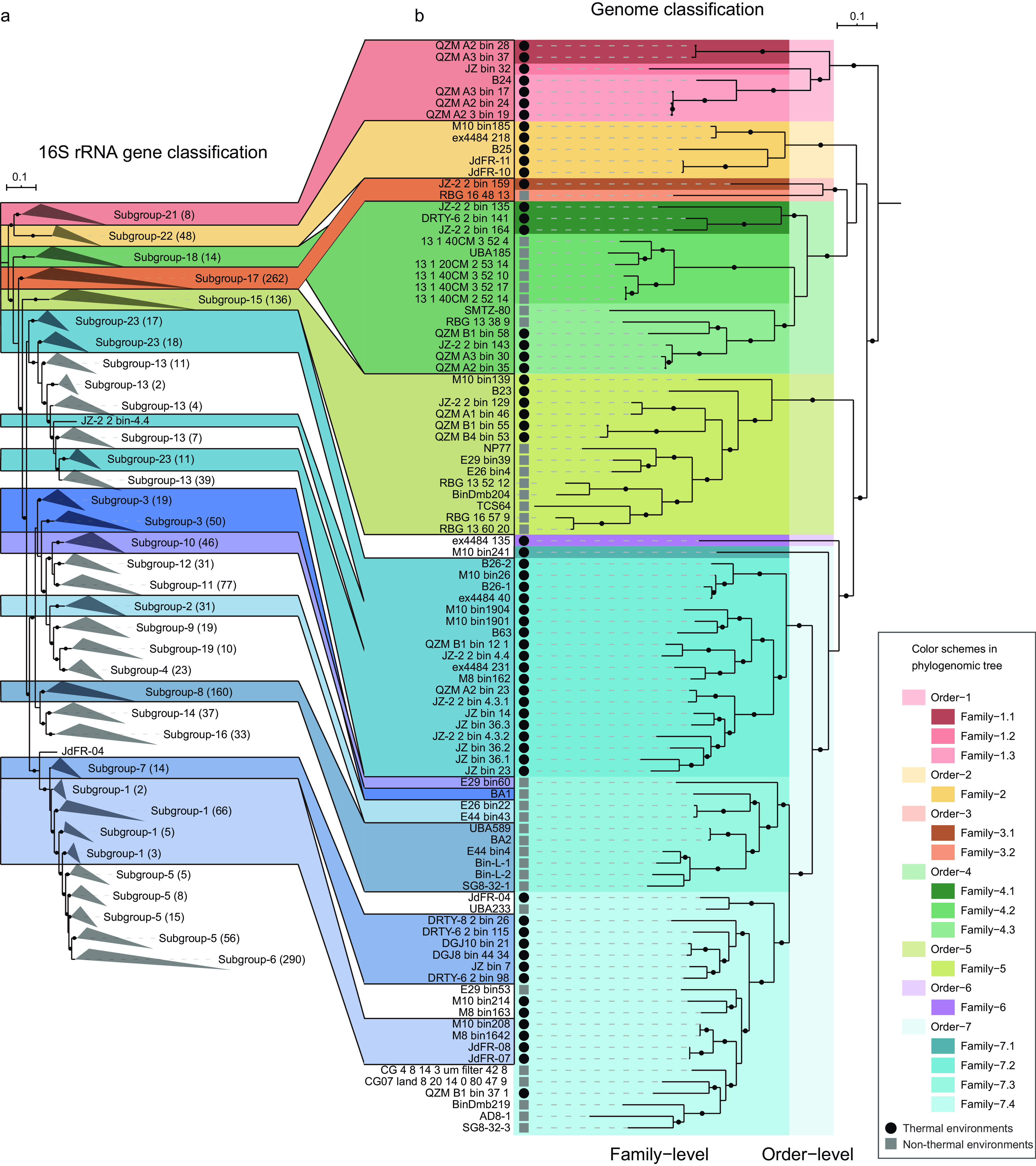
Incongruences between 16S rRNA gene tree and phylogenomic tree of *Ca*. Bathyarchaeia. (a and b) Tanglegram of 16S rRNA gene sequences tree (a) and concatenated 122 archaeal conserved proteins sequences from the 95 *Ca*. Bathyarchaeia MAGs (b). Bootstrap values were calculated from 1,000 iterations using IQ-TREE (see Materials and Methods for details). Bootstrap values of >70% are shown as black circles. The classifications of subgroups in 16S rRNA gene tree are labeled according to Zhou et al. (16) and Feng et al. (22). A total of 1,579 16S rRNA gene sequences are included for the phylogeny reconstruction. Numbers in parentheses describe the number of 16S rRNA gene sequences in the corresponding subgroup. The 16S rRNA-based subgroups without shaded colors, including Subgroup-4, -5, -6, -9, -11, -12, -13, -14, -16, and -19, indicate no representative genomes were detected in these lineages to date.

Studies of *Ca*. Bathyarchaeia diversity relying on the 16S rRNA gene identified up to 23 subgroups (16) (Fig. 1a). In our study, 12 of these 23 subgroups were identified as having representative genomes (Fig. 1a and b), while 13 of the 95 *Ca*. Bathyarchaeia genomes (14%) could not be categorized within the existing subgroups due to the lack of 16S rRNA genes. This is not surprising, as MAGs commonly lack 16S rRNA gene sequences due to assembly technique biases or poor genome completeness (33). While traditionally 16S rRNA-based classification has been useful to classify taxonomic groups such as the *Ca*. Bathyarchaeia, limited metabolic information can be inferred from analysis of this single gene. Therefore, reclassification of this group based on MAGs is necessary. To do this, we used the GTDB genome classification software as an objective measure of taxonomic assignment (38). GTDB is a genome-based taxonomy with phylogenetic consistency that provides rank-normalized classifications for genomes from domain to genus (28), which has previously been missing in the classification of the phylogenetically diverse *Ca*. Bathyarchaeia. By applying this strategy, all 95 *Ca*. Bathyarchaeia MAGs were assigned to seven orders and 15 families (Fig. 1b). Order-1, -2, -3, -4, and -5 were found to correspond to Subgroup-21, -22, -17, -18, and -15, previously utilized by Zhou et al. (16) (Fig. 1a), which had the lowest amino acid identity (AAI) (40% to 51%) between each of the order groupings. The MAG ex4484_135 was found to be the sole representative of the sixth order. The remaining subgroups form the seventh order had the lowest intra-order AAI, at 44%. Of the 35 MAGs newly assembled in this study, they organized into five orders covering nine families (Family-1.1, -1.2, -1.3, -3.1, -4.1, -4.3, -5, -7.2 and -7.4). Six MAGs were assigned to Subgroup-7 and represent the first genomes in this group (Fig. 1b). Likewise, four families, Family-1.1, -1.2, -3.1 and -4.1, represent novel lineages solely represented by MAGs from the Tibet and Tengchong hot springs. An obvious outcome of this comparative analysis was the high level of congruence between the 16S rRNA and GTDB trees (Fig. 1a and b). Apart from the inversion of Order-3 and -4, along with MAGs within Family-7.3, there were little differences between these taxonomies. These results also suggest further work needs to be done to link existing 16S rRNA and MAGs and identify more *Ca*. Bathyarchaeia MAGs and 16S rRNA genes from environmental samples.

### Potential metabolic capabilities of hot spring *Ca*. Bathyarchaeia.

**FIG 2:**
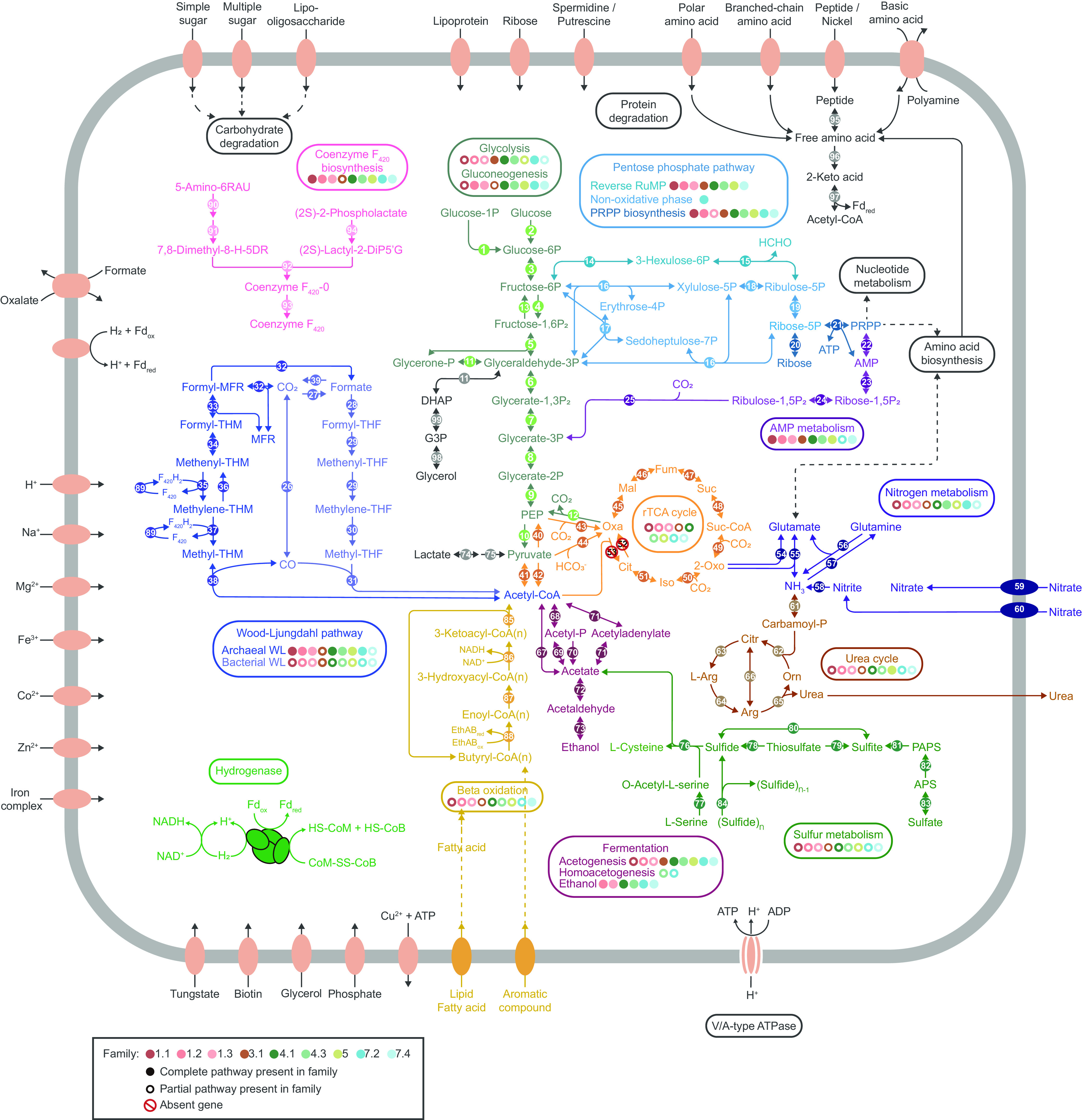
Overview of metabolic potentials in *Ca*. Bathyarchaeia from hot springs. Genes related to glycolysis, gluconeogenesis, the pentose phosphate pathway, AMP metabolism, the Wood-Ljungdahl pathway, the rTCA cycle, nitrogen and sulfur metabolism, the urea cycle, beta-oxidation of fatty acids, fermentation, coenzyme F_420_ biosynthesis, protein degradation, and membrane transporters are shown. Detailed gene copy information associated with above-mentioned pathways is in Data Set S2. Reverse RuMP, reverse ribulose monophosphate pathway; PEP, phosphoenolpyruvate; G3P, glycerol-3-phosphate; DHAP, dihydroxyacetone phosphate; PRPP, phosphoribosyl pyrophosphate; MFR, methanofuran; PAPS, 3′-phosphoadenylyl sulfate; APS, adenylyl sulfate; Fd, ferredoxin.

**FIG 3:**
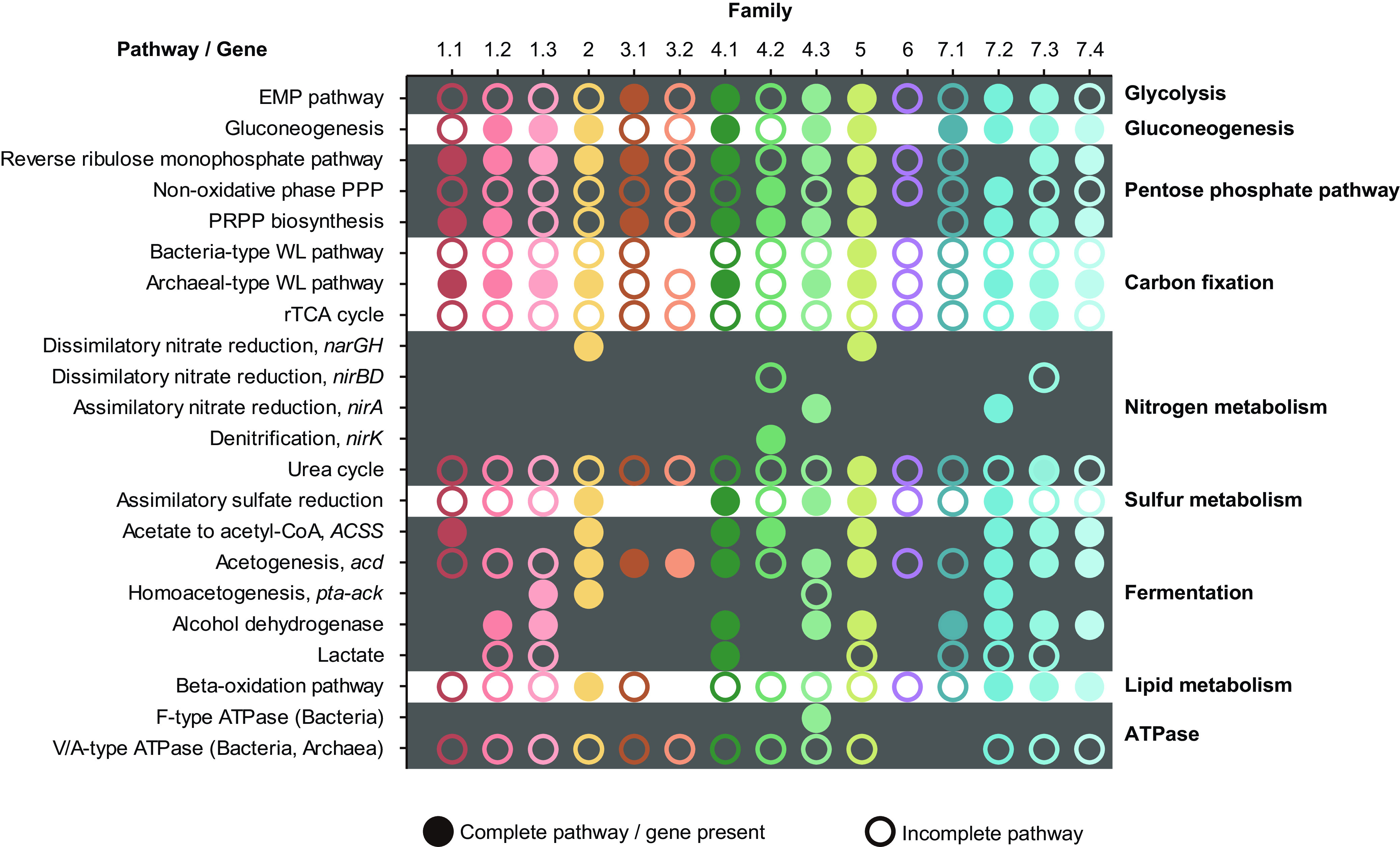
The metabolic properties of 15 *Ca*. Bathyarchaeia families. Solid circles represent the presence of genes or pathways. Hollow circles indicate partial detection of specific pathways. Absence of circles indicates fully missing genes or pathways. PRPP, phosphoribosyl pyrophosphate.

Analysis of open reading frames for central metabolic pathways in the hot spring-associated *Ca*. Bathyarchaeia MAGs Family-3.1, -4.1, and -4.3 revealed the presence of a complete Embden-Meyerhof-Parnas (EMP) glycolysis pathway, whereas other *Ca*. Bathyarchaeia families only contain a partial EMP pathway (Fig. 2, Data Set S2). Genomes in Order-2, -5, and -7 and Family-1.2, -1.3, -4.1, and -4.3 harbor genes for the gluconeogenesis pathway for synthesizing glucose-6-phosphate (or fructose-6-phosphate) from the non-carbohydrate-precursor oxaloacetate (Fig. 3). All the *Ca*. Bathyarchaeia lineages appear to utilize the pentose phosphate pathway (PPP) to generate pentoses and ribose 5-phosphate (R5P), a precursor for the biosynthesis of nucleotides and aromatic amino acids, such as histidine. The MAGs from Family-4.2 and -7.2 have the potential to further convert the R5P into phosphoribosyl pyrophosphate (PRPP) after entering the nonoxidative phase of PPP. However, most other lineages, including Order-1 and -2 and Family-3.1, -4.1, -4.3, -7.3, and -7.4 lack genes for the nonoxidative phase and would likely employ a reverse ribulose monophosphate pathway to perform this same function (39) (Fig. 2 and 3, Data Set S2). Interestingly, Order-5 contains both gene sets, indicating flexibility in the conversion of PRPP and further promoting the synthesis of amino acids and nucleotides (Fig. 3). Consequently, the core metabolic capabilities associated with central carbon processing appear to be similar between these thermophilic *Ca*. Bathyarchaeia MAGs.

10.1128/mSystems.00252-21.8 DATA SET S2 List of genes assigned to metabolic pathways as shown in Fig. 2 of the main text and others. Download Data Set S2, XLS file, 0.2 MB. Copyright © 2021 Qi et al. 2021 Qi et al. https://creativecommons.org/licenses/by/4.0/ This content is distributed under the terms of the Creative Commons Attribution 4.0 International license.

**FIG 4:**
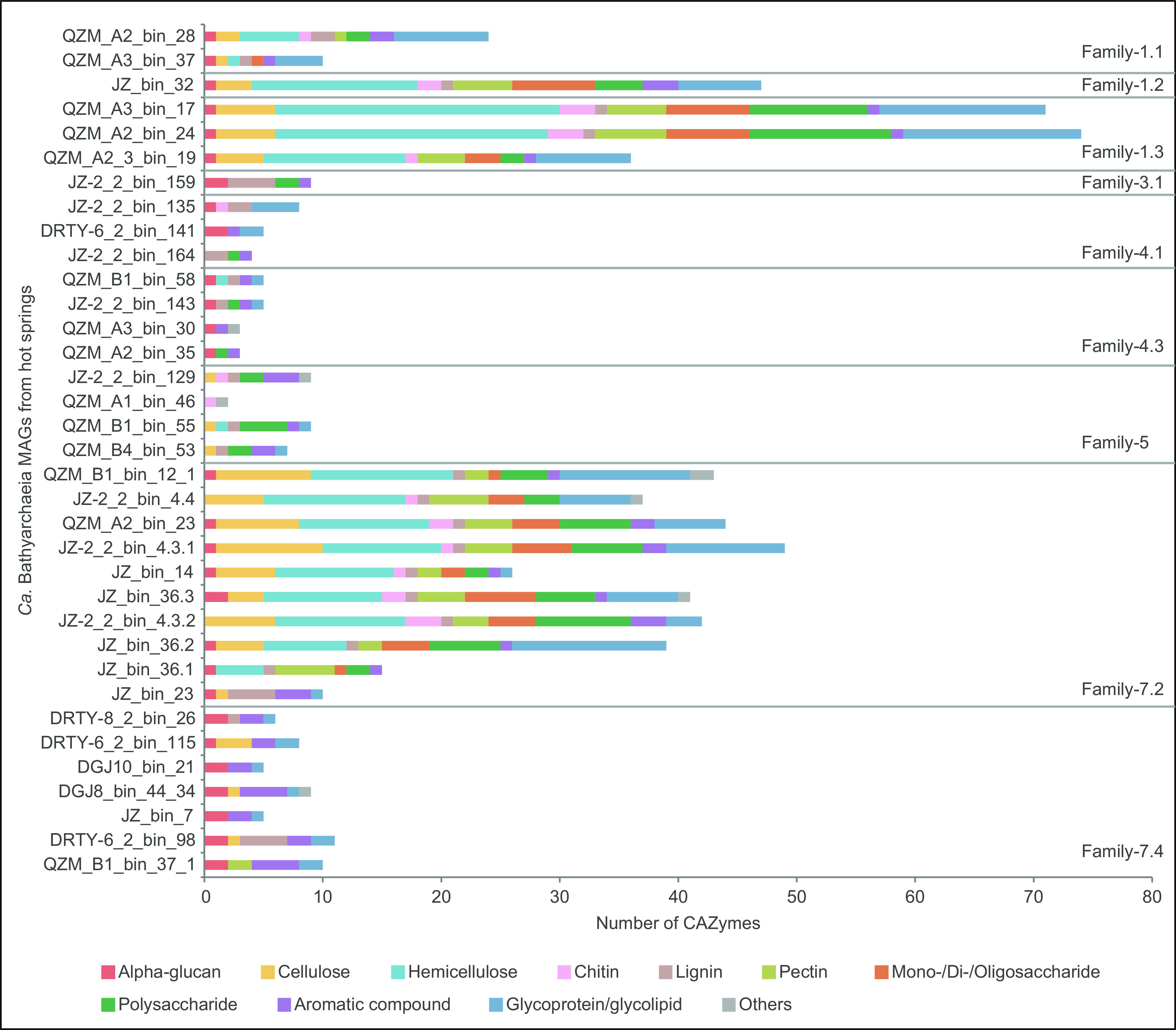
Carbohydrate-active enzymes (CAZymes) detected in 35 *Ca*. Bathyarchaeia MAGs from hot springs.

Beyond carbon flow in central metabolic pathways, the hot springs *Ca*. Bathyarchaeia also appear to metabolize a wide range of carbohydrates. The annotation of carbohydrate-active enzymes (CAZymes) in these MAGs suggested two distinct patterns that correspond to the phylogeny of these organisms (Fig. 4). Family-1.2, -1.3, and -7.2 each contained an unusually high number of CAZymes, ranging from 36 to 74 CAZymes in Family-1.2 and -1.3 MAGs and 10 to 49 CAZymes in Family-7.2 MAGs. In contrast, MAGs in the other families contained 11 or fewer CAZymes, except for the MAG QZM_A2_bin_28 from Family-1.1, which contained 24 CAZymes. This pattern suggests the possible catabolic use of a variety of polysaccharides by members of Family-1.2, -1.3, and -7.2, including alpha-glucan, cellulose, hemicellulose, chitin, lignin, pectin, aromatic compounds, glycoproteins, and glycolipids. The other thermophilic *Ca*. Bathyarchaeia likely consume few, if any, polysaccharides. The ubiquity and abundance of CAZymes in Family-1.2, -1.3, and -7.2 of *Ca*. Bathyarchaeia, and their wide distribution in terrestrial geothermal environments, suggest they may be important primary degraders of complex organic carbon in thermophilic microbial communities. As typical heterotrophs, members of *Ca*. Bathyarchaeia likely generate low-molecular-weight organic compounds to promote the growth of other microorganisms. This would not only facilitate the conversion of biomass to more usable forms, but also would expand ecological niches within their community and allow them persist in these extreme environments (40).

It appears that the *Ca*. Bathyarchaeia MAGs are able to metabolize acetate via two distinct pathways. Specifically, the archaeal-type acetate-forming gene encoded by ADP-forming acetyl-CoA synthetase (*acd*) is found in many lineages, including Order-2, -3, and -5 and Family-4.1, -4.3, -7.2, -7.3, and -7.4 (Fig. 2 and 3). Alternatively, the bacterial-type acetate-forming genes phosphate acetyltransferase (*pta*) and acetate kinase (*ack*) were detected in Family-1.3, -2, and -7.2 MAGs, which are exclusively distributed in hydrothermal vent environments. This result indicates the ability to produce acetate by *Ca*. Bathyarchaeia can be lineage, or at least environment, specific. Also, Family-4.3 from hot springs may have the potential to form acetate, since all four MAGs in this lineage contain the *ack* gene (Data Set S2). *Ca*. Bathyarchaeia utilization of acetate has been demonstrated previously in ^13^C-labeled acetate incubations of estuarine sediments (41–43). Furthermore, the presence of alcohol dehydrogenases among MAGs from Family-1.2, -1.3, -4.1, -4.3, and -5 and Order-7 suggests they may have the ability to ferment other small organic compounds (18) (Fig. 3).

Previously, it has been suggested that *Ca*. Bathyarchaeia perform carbon fixation via bacterial and archaeal type Wood–Ljungdahl (WL) pathways (22). In the 35 *Ca*. Bathyarchaeia MAGs from hot springs, all families contain the key enzyme involved in carbon fixation via the WL pathway, the carbon monoxide dehydrogenase/acetyl-CoA synthase complex (*cdh*/*acs*), which catalyzes the reversible reduction of acetyl-CoA from methyl-tetrahydromethanopterin (Fig. 2, Data Set S2). Seven of nine families (Family-1.1, 1.2, -1.3, -4.1, -4.3, -5, and -7.2) from hot springs possess the complete archaeal-type WL pathway with tetrahydromethanopterin (H_4_MPT) as C_1_-carrier (Fig. 2). The ability to fix carbon dioxide via the WL pathway and form acetate has been predicted previously in *Ca*. Bathyarchaeia and supported by the activity of heterologously expressed *Ca*. Bathyarchaeia acetate kinase *in vitro* (17). The nearly complete archaeal-type of WL pathway in Family-3.1 and -7.4 suggests they may also have the capacity to fix carbon dioxide. However, the 5,10-methylenetetrahydromethanopterin reductase (*mer*) was not recovered in MAGs from these two families. This suggests they may utilize an alternative and unknown complex to perform this same function. Also, by using tetrahydrofolate as C_1_-carrier, some *Ca*. Bathyarchaeia can fix carbon dioxide via the bacterial type of WL pathway (44). In particular, Family-5 harbors all genes necessary for the bacteria-type WL pathway, including the key enzyme formate dehydrogenase (*fdhA*), which converts CO_2_ to formate, with the exception of methylenetetrahydrofolate reductase (*metF*), which is absent (44). *Ca*. Bathyarchaeia are presumably able to take advantage of both WL pathway types to conserve energy, promoting their adaptability to thrive across several environments.

Neither an ATP-citrate lyase nor citrate synthase were detected in any of the 35 hot spring-derived MAGs, suggesting the inability of the tricarboxylic acid cycle (TCA cycle) and reductive tricarboxylic acid cycle (rTCA cycle) to function in *Ca*. Bathyarchaeia. The ribulose 1,5-bisphosphate carboxylase/oxygenase (RuBisCO) gene, an important component of the Calvin-Benson-Bassham (CBB) cycle, was observed in Order-1, Family-3.1, -4.1, -4.3, and -5, but not Family-7.2 and -7.4 (Data Set S2). Phylogenetic analysis showed that most RuBisCO gene sequences affiliated with the form-III type enzymes (Fig. S2), which may be involved in the pathway for AMP metabolism. We rule out the possibility of a carbon fixation pathway using the CBB cycle due to the lack of phosphoribulokinase in all *Ca*. Bathyarchaeia genomes (45) (Data Set S2). The wide detection of genes involved in the AMP metabolism pathway, including adenine phosphoribosyltransferase, AMP phosphorylase, and ribose 1,5-bisphosphate isomerase, gives further supporting evidence for the role of *Ca*. Bathyarchaeia RuBisCO enzymes in the metabolism of AMP. The end product of this AMP pathway, glycerate-3-phosphate (G3P), would then enter the previously mentioned EMP glycolysis pathway.

10.1128/mSystems.00252-21.2 FIG S2 Phylogenetic tree of the ribulose 1,5-bisphosphate carboxylase/oxygenase (RuBisCO). Reference sequences of RuBisCO are selected from Jaffe *et al.* (2019). The alignment of all sequences is conducted using MAFFT. The poorly aligned regions are trimmed by TrimAl with the parameters as: -gt 0.05 -cons 50. The phylogeny was generated using IQ-TREE by iterating 1,000 times, and the best-fit model is LG+F+R10. Download FIG S2, PDF file, 0.8 MB. Copyright © 2021 Qi et al. 2021 Qi et al. https://creativecommons.org/licenses/by/4.0/ This content is distributed under the terms of the Creative Commons Attribution 4.0 International license.

### Comparative genomics of *Ca*. Bathyarchaeia from thermal and nonthermal environments.

**FIG 5:**
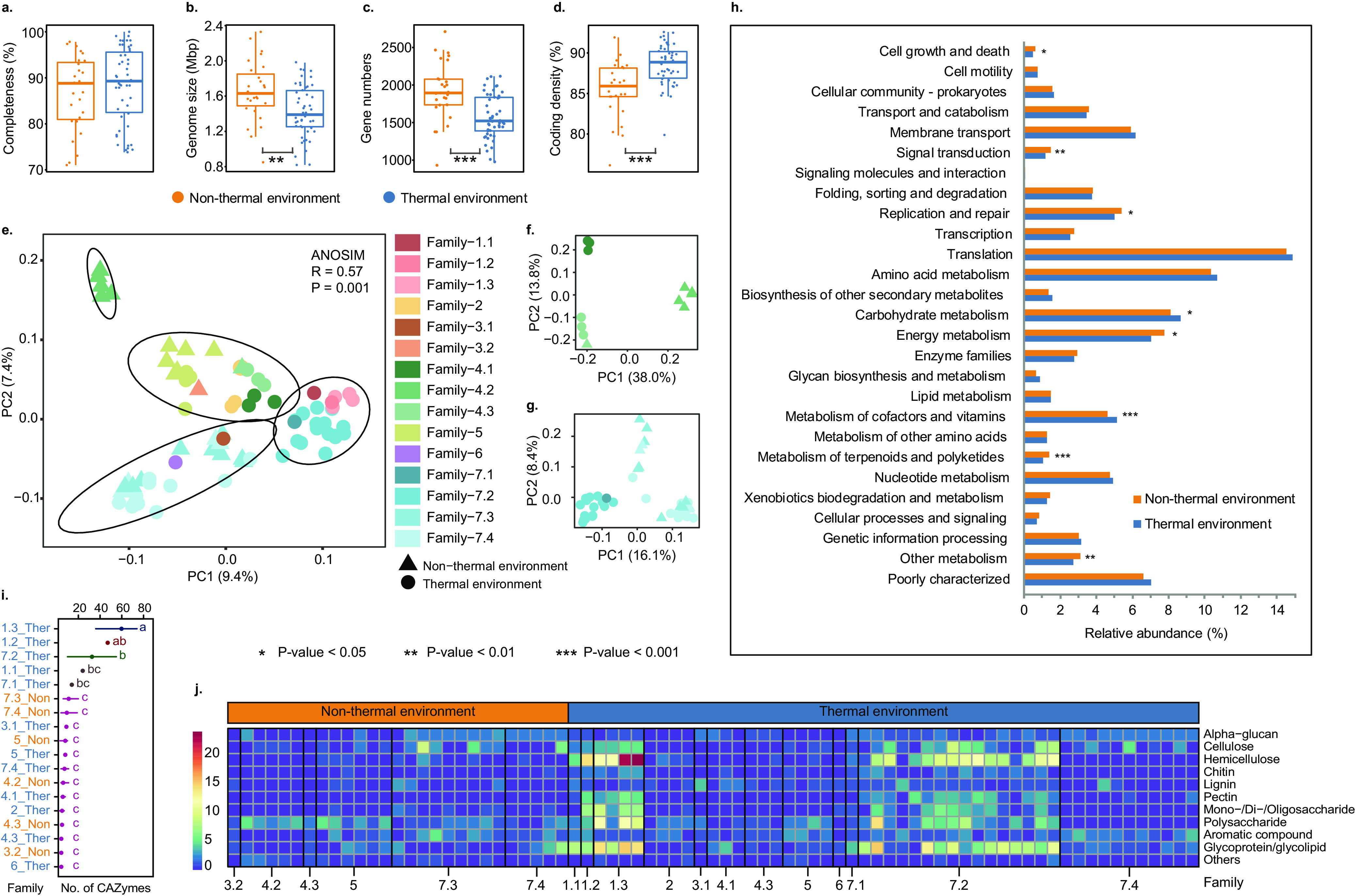
Genomic differences between thermophilic and nonthermophilic *Ca*. Bathyarchaeia genomes. (a to d) Comparison of genome completeness (a), genome sizes (b), gene counts (c), and coding density (d). (e to g) Principal-component analysis (PCA) plots with Euclidean distances based on the functional profiling of all *Ca*. Bathyarchaeia genomes (e), genomes from Order-4 (f), and Order-7 (g) annotated by the KEGG database. (h) Genomic differences at KEGG category level between thermal and nonthermal groups. (i) Comparison of genes related to carbohydrate degradation between taxonomic groups. (j) Heatmap showing the enrichment of genes related to carbohydrate degradation among taxonomic groups.

Although *Ca*. Bathyarchaeia have been reported to have an evolutionary origin in hot environments (22), attributes specific to thermophilic *Ca*. Bathyarchaeia remain unknown. Comparative genomics was leveraged to reveal genomic features that are specific to the thermophilic lineages. The similar completeness of the *Ca*. Bathyarchaeia MAGs from thermal and nonthermal environments, 88.40% and 86.70% mean estimated completeness, respectively, ensures the validity of the comparison between these groups (Fig. 5a). The average genome size of the thermophilic MAGs was 1.42 Mbp and was significantly less than nonthermophilic MAGs at 1.65 Mbp (Mann-Whitney *U* test, *P* = 0.004362; Fig. 5b). Consequently, the thermophilic MAGs on average contained fewer putative open reading frames at 1,578, compared to the nonthermophilic MAGs at 1,918 open reading frames (Mann-Whitney *U* test, *P* = 2.51 × 10^−4^; Fig. 5c). However, this decreased number of open reading frames was slightly offset by a higher coding density in the thermophilic MAGs, of 88.54% compared to 85.55% in the nonthermophilic MAGs (Mann-Whitney *U* test, *P* = 6.68 × 10^−5^; Fig. 5d). A possible explanation for these differences is that thermophilic microorganisms favor small genomes from reduced noncoding regions driven by the so-called genome streamlining process (46–48). A whole-genome comparison of all the 77 MAGs based on KEGG Orthology (KO) showed them clustering into four distinct groups (the ANOSIM test, *R* = 0.57, *P* = 0.001; Fig. 5e). It appears the taxonomic lineage and the habitat type work in concert to shape the clustering pattern of these MAGs (Fig. 5e). Notably, only one of the four KO clusters is solely constituted by MAGs from thermal environments and comprises Family-1.1, -1.2, -1.3, -7.1, and -7.2. While the thermal and nonthermal clusters for the three families of Order-4 were well distinguished based on their phylogenetic relationships (Fig. 5f), thermal and nonthermal MAGs in Family-7.4 were not well separated (Fig. 5g). KEGG Orthology (KO) functional gene profiling revealed significant differences between thermal- and non-thermal-derived MAGs (Fig. 5h), with genes related to cell growth and death, signal transduction, replication and repair, energy metabolism, and metabolism of terpenoids and polyketides being more enriched in nonthermal MAGs (Fig. 5h). In contrast, genes relevant to carbohydrate metabolism are more abundant in the thermal MAGs, which appears to be mainly driven by the previously discussed CAZymes in Family-1.2, -1.3, and -7.2 (Fig. 4).

To further explore the distribution of carbohydrate metabolism capabilities in the *Ca*. Bathyarchaeia MAGs, CAZy-based annotation of these genes was conducted. Consistent with the above results (Fig. 4), the thermal-derived MAGs from Family-1.2, -1.3, and -7.2 harbor a significantly higher number of CAZymes related to carbohydrate degradation compared to nonthermal lineages (least significance difference test, all *P* values < 0.05; Fig. 5i). Specifically, genes associated with the degradation of cellulose (β-glucosidase, endoglucanase), hemicellulose (α-L-fucosidase), pectin (α-L-rhamnosidases), oligosaccharides, and other polysaccharides are more enriched in these three families (Fig. 5j; Data Set S3). Family-1.1 and -7.1, both exclusively from thermal habitats, do show a higher number of CAZymes than most nonthermal lineages, although this result was not significant. The likely ability to utilize a wide range of carbohydrates by Family-1.2, -1.3, and -7.2 is a likely driver for the evolutionary differentiation and may be a vital strategy for survival in thermal environments. Hence, we propose a plausible evolutionary scenario where these thermophilic *Ca*. Bathyarchaeia utilize a variety of polysaccharides as part of a generalized heterotrophic metabolism in nutrient-poor and extreme geothermal environments, as previously suggested for other saccharolytic thermophiles (40).

10.1128/mSystems.00252-21.9 DATA SET S3 List of genes in 77 MAGs involved in degradation of organic carbon compounds, identified by comparison to the CAZy database. Download Data Set S3, XLS file, 0.07 MB. Copyright © 2021 Qi et al. 2021 Qi et al. https://creativecommons.org/licenses/by/4.0/ This content is distributed under the terms of the Creative Commons Attribution 4.0 International license.

**FIG 6:**
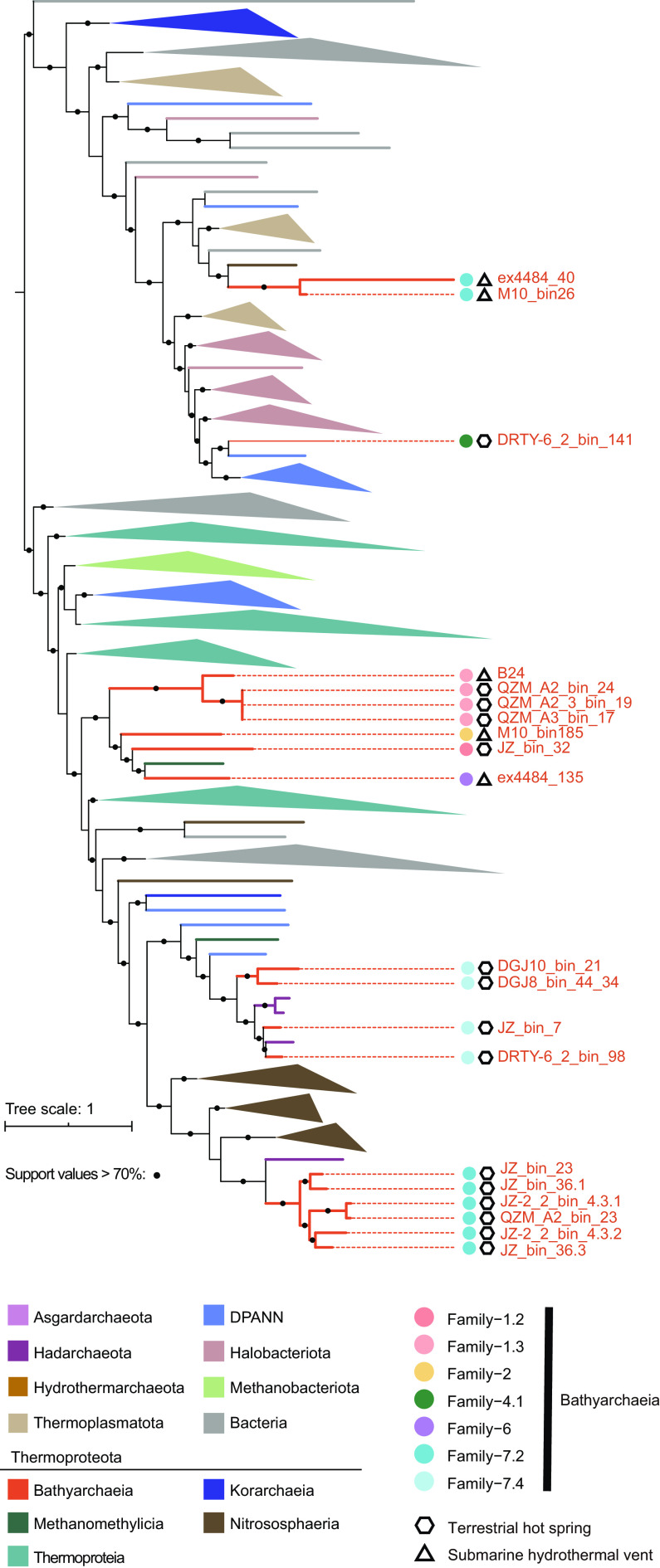
Phylogenetic tree of reverse gyrase (*rgy*). The phylogenetic tree was constructed using IQ-TREE with the best-fit model of LG+F+R10.

While carbohydrate utilization is one mechanism associated with some thermophilic *Ca*. Bathyarchaeia, other thermophilic MAGs also included diverse molecular chaperones, including heat shock proteins (HtpX and Hsp20) and DNA repair enzymes (RadAB) (Fig. S3). These mechanisms are similar to those commonly utilized by other thermophilic microbes to deal with heat stress (49). However, these genes were found across all *Ca*. Bathyarchaeia MAGs, suggesting they were retained from the thermophilic ancestor of the *Ca*. Bathyarchaeia. One key determinant of thermophily is reverse gyrase (*rgy*), which was detected only in thermal-derived bathyarchaeial families, including Family-1.2, -1.3, -2, -4.1, -6, -7.2, and -7.4. Further phylogenetic analysis reveals a complicated evolutionary history of the *rgy* gene, with frequent HGTs detected (Fig. 6). It is likely that *Ca*. Bathyarchaeia MAGs containing the *rgy* genes are obligate thermophilic organisms. This result is further supported by the absence of the DnaK-DnaJ-GrpE chaperone system in the thermal *Ca*. Bathyarchaeia, as this system is considered to be important to mesophiles (50). The widespread presence of the DnaK-DnaJ-GrpE genes in mesophilic MAGs and in those thermophilic MAGs without *rgy* suggests this may be the case. Interestingly, two genomes (M10_bin185 and DRTY-6_2_bin_141) contain both the *rgy* gene and DnaK-DnaJ-GrpE chaperone system, suggesting they may have the ability to grow and/or survive in a wider range of temperatures.

10.1128/mSystems.00252-21.3 FIG S3 Heat-related genes of *Ca*. Bathyarchaeia MAGs in thermal and nonthermal environments. Solid circles indicate the presence of this gene. Download FIG S3, PDF file, 0.8 MB. Copyright © 2021 Qi et al. 2021 Qi et al. https://creativecommons.org/licenses/by/4.0/ This content is distributed under the terms of the Creative Commons Attribution 4.0 International license.

### Evolution of methane and alkane metabolism in *Ca*. Bathyarchaeia.

While many *Ca*. Bathyarchaeia appear to be heterotrophs, two *Ca*. Bathyarchaeia MAGs were recently shown to possess genes for the methyl-coenzyme M reductase (*mcrABG*) complex, a key enzyme involved in methane/alkane metabolism. These two MAGs were the first archaea outside of the classic methanogen/methanotroph lineages to be identified with these genes (23). Their Mcr complexes fall into a cluster that also contains *mcr* genes from the *Halobacteriota* (per GTDB), including *Ca.* Syntrophoarchaeum (25), *Archaeoglobi* (51), *Ca*. Methanoliparia (52), and *Ca*. Hadarchaeota (27, 53), along with members from the *Ca*. Helarchaeales (54). Phylogenetic analysis places all of these *mcrABG* sequences into a lineage distant from traditional archaeal methanogens and methanotrophs (Fig. S4) and suggests they are involved in the oxidation of short chain alkanes such as butane/propane, rather than methane metabolism (25). While it has been suggested that these *Ca*. Bathyarchaeia may conduct alkane oxidation, the detection of genes for *β*-oxidation and acetyl-CoA oxidation pathways suggest these *mcr*-containing *Ca*. Bathyarchaeia may have metabolic capabilities similar to those proposed for the alkane-oxidizing *Ca.* Syntrophoarchaeum (25). Given the multiple copies of Mcr genes in *Ca*. Syntrophoarchaeum, and phylogenetically diverse taxa containing related Mcr complexes, we speculate that the Mcr complexes in *Ca*. Bathyarchaeia, *Ca*. Hadarchaeota, and *Ca*. Helarchaeales are likely to have been derived from *Ca.* Syntrophoarchaeum via HGT events (Fig. S4). This result would be consistent with other suggestions that HGT is a driver of the transfer of this gene complex (54).

10.1128/mSystems.00252-21.4 FIG S4 Phylogenetic tree of concatenated *mcrABG* genes. Reference amino acid sequence, including methanogens, methanotrohps, and alkanotrophs, were collected in previous studies. The triangle mark indicates that ethane (red) and butane and propane (green) oxidation has been experimentally verified. The best model for the phylogenetic tree is LG+F+R6. Download FIG S4, PDF file, 0.2 MB. Copyright © 2021 Qi et al. 2021 Qi et al. https://creativecommons.org/licenses/by/4.0/ This content is distributed under the terms of the Creative Commons Attribution 4.0 International license.

**FIG 7:**
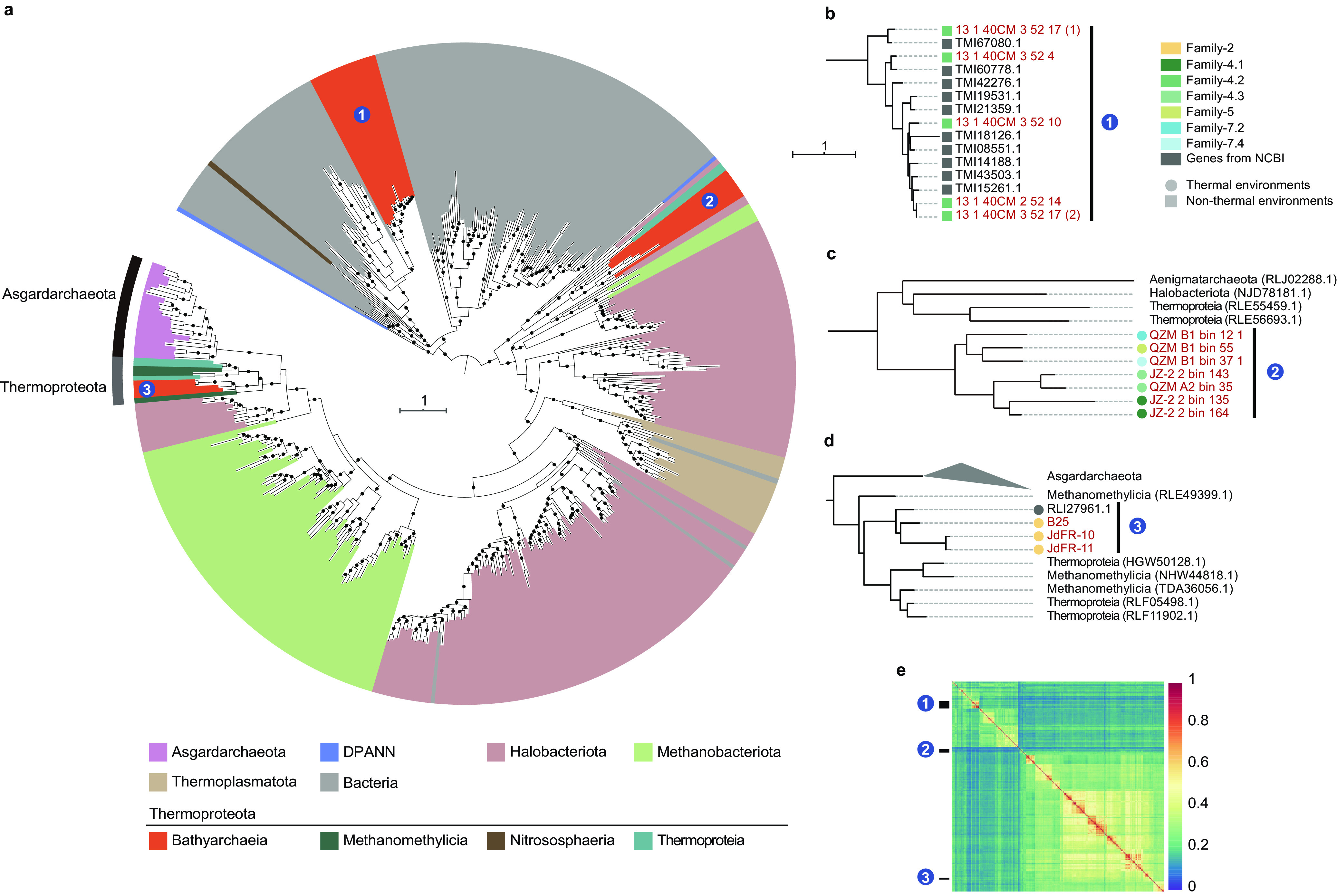
The phylogeny of tetrahydromethanopterin *S*-methyltransferase subunit A (*mtrA*) gene. (a) Maximum-likelihood tree of the *mtrA* gene. The phylogenetic tree was constructed using IQ-TREE with WAG+F+R8 as the best model. Support values of >70% are shown as black circles. (b to d) Sublineages of the phylogenetic tree which contain *mtrA* genes from *Ca*. Bathyarchaeia. (e) Sequence identities between each pair of *mtrA* gene sequences.

Along with the Mcr complex, a variety of other methanogenesis-related genes were detected in the *Ca*. Bathyarchaeia MAGs. Most methanogens encode the membrane-bound tetrahydromethanopterin S-methyltransferase (*mtrABCDEFGH*) complex, catalyzing the energy-conserving (Na^+^-translocating) methyl transfer from methyltetrahydromethanopterin (H_4_MPT) to coenzyme M (CoM-SH). For the *Ca*. Bathyarchaeia, Family-2 appears to be the only lineage that possesses genes that would encode a nearly complete MTR complex (Data Set S4), and the reason for their presence in these MAGs remains unclear. While *mtrAH* genes were detected in 6 of 35 terrestrial thermophilic *Ca*. Bathyarchaeia, it has been reported previously that *mtrAH* genes in *Nitrososphaeria* would likely encode for corrinoid and methyltransferase proteins that would allow for the assimilation of unknown methylated compounds (23, 27, 55). Given the widespread nature of these *mtrAH* genes in the *Ca*. Bathyarchaeia, phylogenetic trees of *mtrA* genes were generated to better understand their evolution (Fig. 7a). This analysis showed that *mtrA* genes from nonthermophilic *Ca*. Bathyarchaeia clustered into a single group, suggesting an ancient HGT event with the bacterial phylum *Actinobacteriota* as the potential donor (Fig. 7b). Alternatively, the thermophilic *Ca*. Bathyarchaeia *mtrA* sequences form two separate clusters and appear to have two independent evolutionary histories (Fig. 7). Likewise, cluster 3 from the thermophilic lineages is relatively conserved, though their ancestor may have been transferred from *Halobacteriota* or *Methanobacteriota* (Fig. 7d). While this result appears clear, the presence of *Asgardarchaeota mtrA* genes with low bootstrap values suggests the placements of these *Ca*. Bathyarchaeia *mtrA* sequences may change as additional sequences are discovered. No obvious HGTs are detected for the ancestor of the *mtrA* genes derived from terrestrial thermal habitats in cluster 2, given that *Thermoproteia* sequences form a sister lineage (Fig. 7c). It also needs to be mentioned that the *Ca*. Bathyarchaeia from Family-2 are exclusively from hydrothermal habitats and again suggests thermophilic *mtrA* genes from terrestrial (cluster 2) and marine (cluster 3) ecosystems have different evolutionary histories.

10.1128/mSystems.00252-21.10 DATA SET S4 Alkane metabolism-related genes in 15 families of *Ca*. Bathyarchaeia. Download Data Set S4, XLS file, 0.06 MB. Copyright © 2021 Qi et al. 2021 Qi et al. https://creativecommons.org/licenses/by/4.0/ This content is distributed under the terms of the Creative Commons Attribution 4.0 International license.

**FIG 8:**
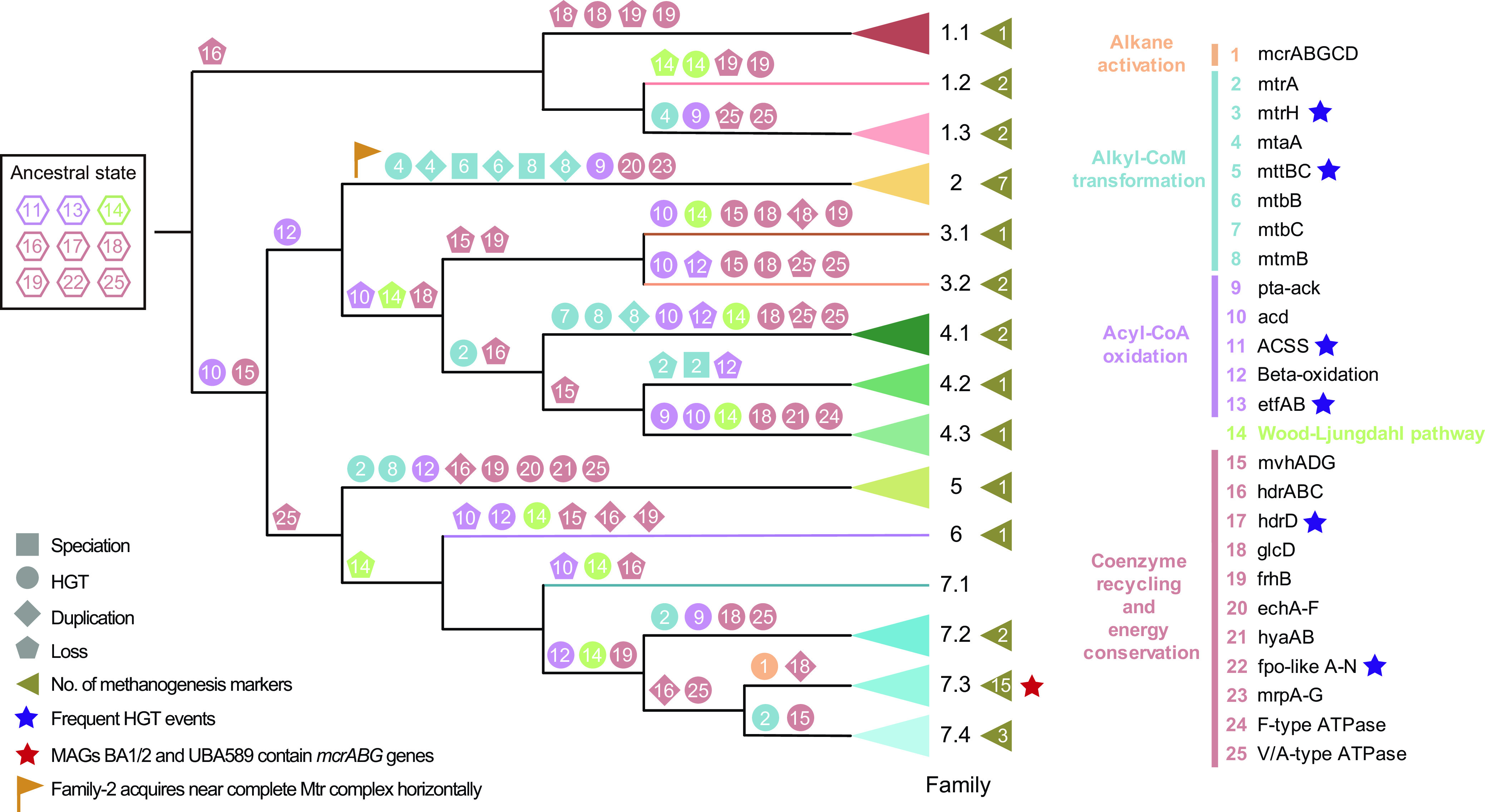
Evolutionary history reconstruction of methane/alkane metabolism in *Ca*. Bathyarchaeia. The cladogram shows the phylogenetic position of all genomes from *Ca*. Bathyarchaeia, which is the same as in Fig. 1. Families with more than one genome are collapsed. Different shapes on each node depict different evolutionary events, including speciation, duplication, HGT, and loss; colors represent genes from different functional modules. The numbers of methanogenesis marker genes in each family are shown in left-facing triangles adjacent to family numbers.

Amalgamated likelihood estimation (ALE) analyses consolidate the inference that a speciation event, rather than other gene acquisition, occurred for *mtrA* genes in Family-4.2 (Fig. 8). These results suggest the *mtrA* gene homologs in Order-4 have a thermal origin in the ancestor of Order-4 and may have acquired this gene horizontally, while it is preserved in Family-4.1 and -4.3 but lost in Family-4.2. Furthermore, to sustain the ability to metabolize unknown methylated substrates, Family-4.2 *mtrA* genes evolved to function in nonthermal habitats. While the *Ca*. Bathyarchaeia in nonthermal environments and terrestrial hot springs contain genes for a partial Mtr complex (*mtrAH*), the *Ca*. Bathyarchaeia in submarine hydrothermal vents harbor genes that would generate a nearly complete *Mtr* complex. Furthermore, sequence identities among all *mtrA* genes confirm the independent evolutionary trajectories among the three groups (Fig. 7e). In contrast to the clear evolutionary pattern for *mtrA* genes, the *mtrH* subunit shows a much more complicated evolutionary history, where HGT signals are frequently observed among different lineages (Fig. S5).

10.1128/mSystems.00252-21.5 FIG S5 Phylogenetic tree of tetrahydromethanopterin *S*-methyltransferase subunit H (*mtrH*). The best model for the phylogeny is LG+R8. Download FIG S5, PDF file, 0.5 MB. Copyright © 2021 Qi et al. 2021 Qi et al. https://creativecommons.org/licenses/by/4.0/ This content is distributed under the terms of the Creative Commons Attribution 4.0 International license.

To better understand the origin and evolution of the suggested alkane metabolism in *Ca*. Bathyarchaeia, all related genes were recruited into an ALE analysis. Results suggest that the *Ca*. Bathyarchaeia ancestor may have synthesized acetyl-CoA from acetate, fixed carbon dioxide via the WL pathway, and harbored many genes for electron transport and energy conservation, including *etfAB*, *hdrABC*, *hdrD*, *glcD*, *frhB*, *fpo*-like complex genes, and V/A-type ATPase genes (Fig. 8). This analysis also suggests the ancestor did not have the ability to oxidize alkanes due to the lack of the Mcr complex, which is consistent with the inference that it was a HGT event from a *Ca.* Syntrophoarchaeum or similar alkane-oxidizing archaeon into the Family-7.3 MAGs (Fig. 8). Not surprisingly, this *Ca*. Bathyarchaeia lineage contains 24 of the 38 methanogenesis marker proteins that have been reported previously (52). Likewise, there is a clear pattern from the ALE analysis that shows the ancestor of all *Ca*. Bathyarchaeia was unable to metabolize methylated compounds, such as those for methanol and (di/tri)-methylamine utilization via *mtaA*, *mtbB*, *mtmB*, and *mttBC* genes in Family-2 (Data Set S4), and have only been picked up by certain lineages. Also, it is likely that gene duplication of these methyl group utilization genes is another driver of metabolic diversity. For the acyl-CoA oxidation module, *β*-oxidation and acetate production via the *acd* gene or *pta*-*ack* pathway have been acquired via HGT, with the *pta*-*ack* pathway appearing to have only been acquired by MAGs from hydrothermal vent environments (Fig. 8). *β*-Oxidation appears in many lineages, including Family-7.3, which is consistent with the function of Family-7.3 predicted to perform butane/propane oxidation using a similar mechanism to that suggest in *Ca*. Syntrophoarchaeum (25). However, this remains unproven due to the lack of experimental confirmation. Taken together, these results suggest that the ability of Family-7.3 MAGs to potentially oxidize alkanes is the result of HGT rather than vertical decent from a common ancestor. However, we still cannot rule out the possibility that the common ancestor of Family-7.3 and Family-2 may have had this ability, due to the prevalence of genes for alkyl-CoA transformation described previously (53) and several methanogenesis marker genes (Fig. 8) in Family-2.

**FIG 9:**
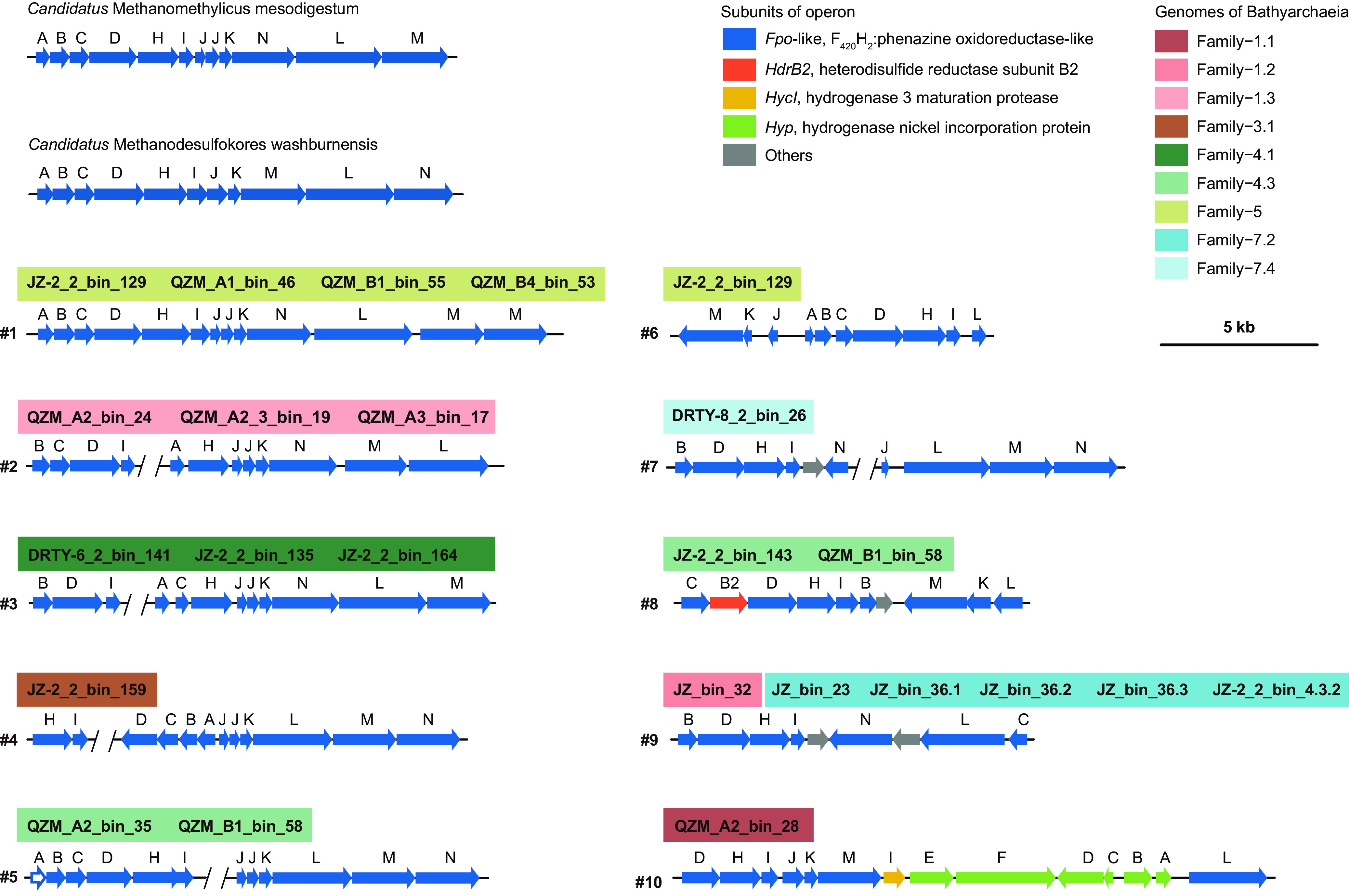
Schematic representation of gene clusters of *fpo*-like complexes in *Ca*. Bathyarchaeia MAGs from hot springs. The subunit A, represented by the hollow arrow in #5 indicates that this subunit should exist but was not detected due to the low sequence quality.

**FIG 10:**
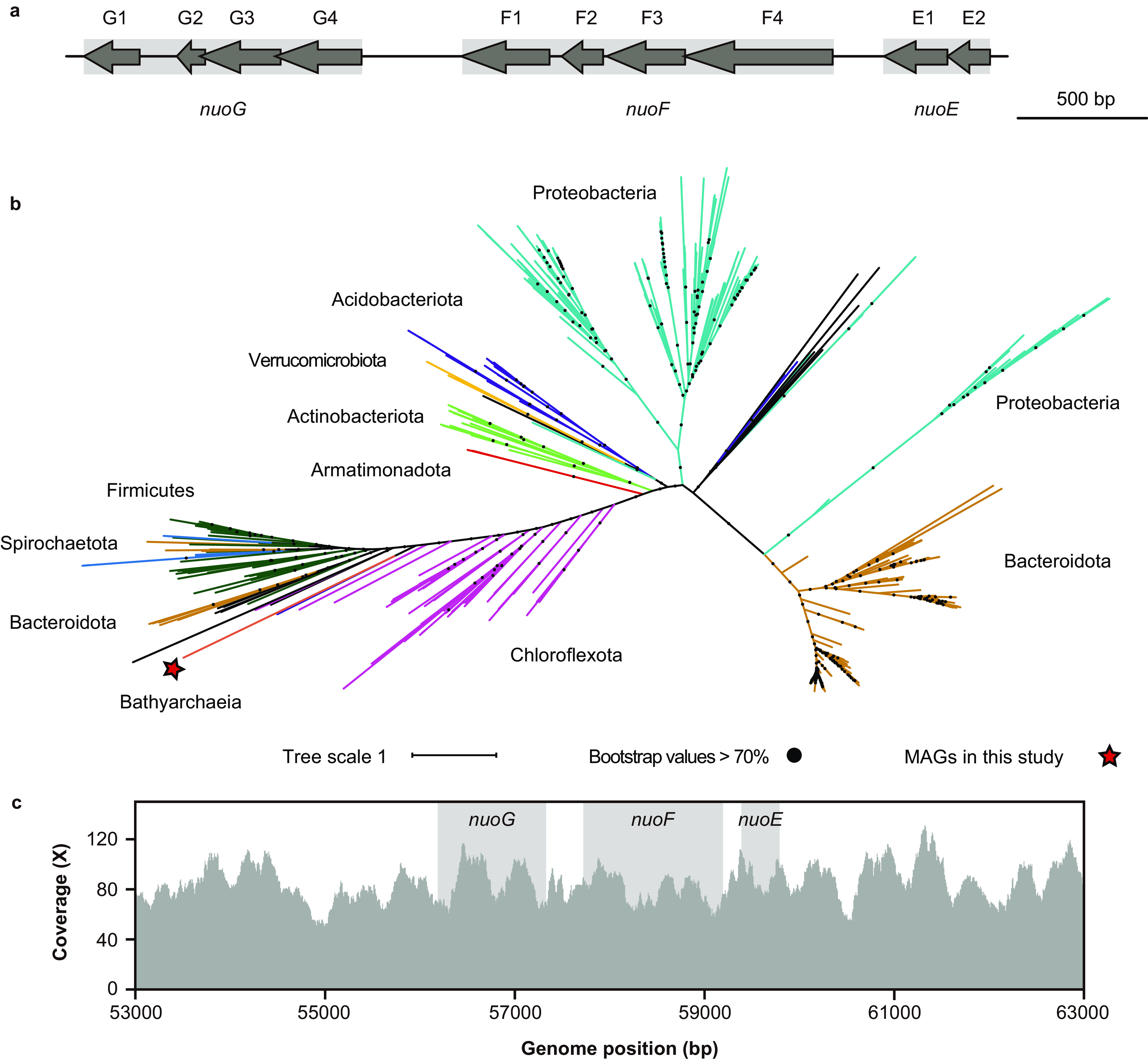
Identified NADH:quinone oxidoreductase subunits EFG (*nuoEFG*) in JZ_bin_32. (a) Schematic representation depicting the gene organizations of the three genes in JZ_bin_32. Arrows represent predicted genes and gene fragmentation is observed in all three genes. Rectangles in gray indicate putative complete genes. (b) Maximum-likelihood phylogenetic tree constructed based on concatenation of *nuoEFG* genes. Bootstrap values are calculated with 1,000 replicates. (c) Histogram showing the sequence depth of each base in the *nuoEFG* genes and nearby regions of the scaffold.

Regarding the coenzyme recycling and energy conservation module, most genes undergo frequent HGT, accompanied by substantial gene duplication events. For example, both *ech* and *mrp* complexes in Family-2 are acquired horizontally, providing sufficient energy for the conversion of methylated compounds (Fig. 8). Similar to *Methanomethylicia* (24) (formerly *Ca*. Verstraetearchaeota phylum) and *Korarchaeia* (52) (formerly *Ca*. Korarchaeota phylum), *Ca*. Bathyarchaeia harbor F_420_H_2_:phenazine oxidoreductase (*fpo*), but lack the *fpoFO* subunits (Data Set S4), suggesting the inability to reoxidize reduced F_420_ for energy conservation seen in some methanogens (56). Instead, they may employ the membrane-bound heterodisulfide reductase subunit D (*hdrD*) to form an energy-converting ferredoxin:heterodisulfide oxidoreductase, and concomitantly to generate a proton motive force across the cytoplasmic membrane; this mechanism has been predicted previously in H_2_-dependent methylotrophic methanogens (57). A total of 22 MAGs within nine families among the 35 MAGs from hot spring sediments harbor the *fpo*-like complex and different families show quite divergent cluster topologies (Fig. 9). Family-4.3 and -5 show similar *fpo* operon structures as predicted methanogens from the *Methanomethylicia* and *Korarchaeia*, except for the insertion of a *fpoM* copy in the operon from Family-5 (#1 and #5 in Fig. 9). Family-1.3, -3.1, and -4.1 contain all subunits but with rearranged operon structures (#2 to #4). Also, genome reduction in the thermophilic MAGs described previously (Fig. 5c) may play a role in some *fpo* operons, as Family-1.1, -1.2, -4.3, -5, -7.2, and -7.4 appear to have lost at least one subunit from this operon (#6 to #10). Conversely, subunits *fpoBDHL* may likely be indispensable (58), as they were always present (Fig. 9). Non-*fpo* genes were also identified within this operon, where *hdrB2* was found in Family-4.3 and suggests alternative sources of electron transfer, such as the reoxidation of coenzyme M-coenzyme B heterodisulfide bonds (CoM-S-S-CoB) (#8) (59). Also, we observed the *nuoEFG* genes in only one (JZ_bin_32) of the 35 thermal MAGs, which may allow this complex to bind and oxidize NADH to NAD^+^ rather than electron carriers such as F_420_, ferredoxin, or CoM-S-S-CoB predicted in other operons (Fig. 10a). While phylogenetic analyses of these *nuoEFG* genes place them within the phylum *Chloroflexota* and suggest these genes may have been obtained via HGT (Fig. 10b), these subunits are fragmented and contain several termination codons, suggesting that these genes may not be functional. Interestingly, the sequence coverage depth of the *nuoEFG*-containing scaffold is high at ∼80× across the assembled scaffold (Fig. 10c).

In conclusion, the present study taken as a whole largely expands the current diversity of *Ca*. Bathyarchaeia MAGs and shows that many of the hot spring-associated lineages are able to fix carbon dioxide and heterotrophically degrade a variety of carbohydrates. Also, it appears that these thermophilic *Ca*. Bathyarchaeia MAGs have evolved a greater number of genes related to carbohydrate degradation and their genomes have undergone genome streamlining consistent with these environments. Furthermore, we show that two lineages may have the ability to metabolize methane/alkane due to the wide detection of genes related to methanogenesis and/or alkane oxidation. Evidence also shows that the acquisition of these metabolic capabilities is likely the result of HGT rather than vertical inheritance. Overall, this study largely expands the understanding of metabolic capacities and evolution of *Ca*. Bathyarchaeia lineages, providing clues for the further discovery of lineages and future isolation of this widespread archaea, while shedding light on the metabolic capabilities of early life on earth.

## MATERIALS AND METHODS

### Sample site, DNA extraction, and metagenomic sequencing.

One hot spring sediment sample JZ was obtained in 2016 and three hot spring sediment samples, including JZ-2_2, DRTY-6_2, and DRTY-8_2 were obtained in 2017 from Tengchong, Yunnan, China. Eight hot spring sediment samples, including DGJ8, DGJ10, QZM_A1, QZM_A2, QZM_A2_3, QZM_A3, QZM_B1, and QZM_B4 were obtained in 2016 from Tibet, China. Sample JZ and JZ-2_2 came from the same place, JinZe Hot Spring Resort in DianTan county (25°26 N, 98°28 E); samples DRTY-6_2 and DRTY-8_2 came from two different pools of DiReTiYan Zone in Rehai Geothermal National Park (24°57 N, 98°26 E); sample DGJ8 and DGJ10 came from two different pools of DaGeJia ChangMaQu east coast in AngDa county (29°36 N, 85°45 E); samples QZM_A1, QZM_A2, QZM_A2_3, QZM_A3, QZM_B1, and QZM_B4 came from six different pools of QuZhuoMu village in CuoNa county (28°15 N, 91°49 E). These samples span a wide range of temperature from 56.9 to 83.0°C and pH values ranging from 6.0 to 7.6. The sample collection, DNA extraction, and metagenomic sequencing are described in a previous study (60).

### Metagenomic assembly and genome binning.

Raw metagenomic reads were generated on an Illumina Hiseq 4000 sequencer were quality filtered to obtain quality reads as described previously (61). Clean reads from each sample were assembled independently using SPAdes v3.9.0 (62) with the following parameters: -k 21,33,55,77,99,127 –meta. The assembled scaffolds with lengths of  >2,500bp were kept for further analysis. Genome binning was conducted using Metabat v2.12.1 (63) and ESOM (Emergent Self-Organizing map) v1.1 (64). Specifically, sequence depth was calculated by mapping quality reads from each sample to the assembled scaffolds separately using BBMap v38.85 (http://sourceforge.net/projects/bbmap/) with parameters as follows: k = 15 minid = 0.9 build = 1. MAGs were generated using Metabat by considering both the sequence depth and tetranucleotide frequency (TNF) information. The genome completeness, contamination, and strain heterogeneity of each MAG were evaluated using CheckM v1.0.5 (65). Scaffolds from all MAGs were sheared into short fragments (5 to 10 kb) and were visualized using ESOM based on their TNF. Low-quality MAGs were manually investigated and scaffolds with abnormal coverage information and discordant positions in the ESOM map were removed, as previously described (27). Finally, cleaned reads for each MAG were recruited using BBMap (the same parameters as mentioned above) and were reassembled using SPAdes with the following parameters: –careful -k 21,33,55,77,99,127. A total of 35 genome bins belonging to *Ca*. Bathyarchaeia were obtained from this process for further analysis.

### Functional annotation of genome bins.

Gene calling for each MAG was conducted using Prodigal v2.6.3 (66) with the “-p single” option. Functional annotation was determined by comparing predicted genes against the National Center for Biotechnology Information (NCBI) nonredundant (nr), Kyoto Encyclopedia of Genes and Genomes (KEGG) (67), Archaeal Clusters of Orthologous Genes (arCOG) (68), and the Pfam protein families (Pfam) databases (69) using DIAMOND v0.8.22.84 (70) with E value of <1e^−5^. All predicted open reading frames were searched against the dbCAN2 (71) meta server v9 online to find homologs associated with the degradation of carbohydrates. The carbohydrate-degrading enzymes were further classified using the carbohydrate-active enzymes database (72) and CAZypedia (73). The utilized substrates linked with specific CAZY genes were reported in previous published literature (74–81).

### Phylogenetic analysis.

In total, 95 *Ca*. Bathyarchaeia MAGs including 35 from this study and 60 from currently available public databases (NCBI refSeq and IMG databases, downloaded 7 June 2019) with more than 50% genomic completeness and less than 10% genomic contamination were collected for the phylogenomic tree reconstruction. The 95 *Ca*. Bathyarchaeia MAGs were incorporated into the GTDB-tk v0.2.2 (82), an open-source toolkit for the taxonomic classification of genome and MAG assemblies with 122 concatenated archaeal single-copy marker protein sequences. The concatenated alignment was used to generate phylogeny by applying IQ-TREE v1.6.10 (83) with the mixture model of LG+F+R8 and with 1,000 ultrafast bootstrapping. The best model was determined by ModelFinder (84), which is well supported by Bayesian information criterion (BIC).

A phylogenetic tree based on 16S rRNA gene was generated using RNAmmer v1.2 (85) to identify nearly complete 16S rRNA genes in the 95 *Ca*. Bathyarchaeia genomes, with the parameters as following: -S arc -multi -m ssu. MAG DNA sequences were searched using the BLASTn program (86) against RDP database (87) (downloaded 18 October 2018) to detect partial 16S rRNA genes not detected by RNAmmer. Only sequences with lengths >300 bp were taken into consideration. The 16S rRNA gene sequences of 23 *Ca*. Bathyarchaeia subgroups classified by Zhou et al. (16) and Feng et al. (22) were used as phylogenetic anchors. All sequences were aligned together using the MAFFT v6.864b (88) online server with the strategy as follows: iterative refinement method “FFT -NS –I.” The poorly aligned regions were trimmed by TrimAl v1.4.rev22 (89) with the parameters as follows: -gt 0.05 -cons 50. The 16S rRNA gene phylogeny was generated using IQ-TREE by iterating 1,000 times and the best-fit model was SYM+R10.

For the gene taxonomies of *mtrA*, *rgy*, *mtrH,* and *nuoEFG*, amino acid sequences were downloaded from NCBI by applying BLAST searches with the corresponding sequences from present *Ca*. Bathyarchaeia MAGs as inputs. Gene sequences of *mcrABG* from previous studies were used for analyses (27). Individual genes were aligned using MUSCLE v3.8.31 (90) by iterating 100 times. Gene complexes, such as *nuoEFG* and *mcrABG*, were concatenated. Poorly aligned regions were eliminated using TrimAl with the same parameters as above. Phylogenies were reconstructed using IQ-TREE with ultrafast bootstrapping (-bb 1000), as well as Shimodaira-Hasegawa-like approximate likelihood-ratio test (SH-aLRT, -alrt 1000). The best models are reported in the corresponding figure legends.

All phylogenetic trees were visualized using iTOL v3 (91).

### Comparative genomics between thermal environments and nonthermal environments.

Genomes with completeness of ≥70% were picked for comparative genomics analysis, resulting in 77 of the 95 *Ca*. Bathyarchaeia MAGs being selected, and were further classified into thermal and nonthermal groups based on their habitat temperature. PCA clustering analyses based on Euclidean distances were performed for selected genomes annotated with the KEGG database using vegan package v2.5-6 (92). Significant differences among groups were examined using the analysis of similarities (ANOSIM). The differences of relative abundances of KEGG categories were examined between two groups using the Wilcoxon rank-sum test. All *P* values were adjusted using the “BH” correction in R. The comparisons of CAZyme number in each *Ca*. Bathyarchaeia family pair was analyzed by the least significant difference (LSD) test.

### Evolutionary history reconstruction of alkane and methane metabolism.

Genes related to alkane activation, alkyl-CoM transformation, acyl-CoA oxidation, carbon fixation via Wood-Ljungdahl pathway, and coenzyme cycling and energy conservation were selected for the present analysis. Putative protein-coding sequences were picked from all 95 *Ca*. Bathyarchaeia genomes and aligned using MUSCLE. Poorly aligned regions were eliminated using TrimAl and phylogenies of selected genes were generated using IQ-TREE. To infer the potential evolution scenarios of gene gain, duplication, transfer, and loss (DTL), amalgamated likelihood estimation (ALE) was used to calculate the likelihood for each of the 98 gene families encoded by 95 *Ca*. Bathyarchaeia genomes. The species tree was constructed on a concatenation of 122 conserved single-copy genes, as described above. The rates of DTL were inferred from the data using maximum likelihood optimization and reconciliations between gene trees and species tree were conducted using the *ALEml_undated* algorithm in the ALE package (93).

### Data availability.

Metagenome-assembled genomes described in this study have been deposited to NCBI under the BioProject PRJNA544494: BioSample id SAMN18244059, SAMN18244060, SAMN18253264, SAMN18253267, SAMN18253270, SAMN18838809 to SAMN18838815, and the accession numbers are JAGTQA000000000 to JAGTQZ000000000, JAGTRA000000000 to JAGTRI000000000. The data sets generated during and/or analyzed during the current study are available from the corresponding author upon reasonable request.

## References

[1] Barns SM, Delwiche CF, Palmer JD, Pace NR. 1996. Perspectives on archaeal diversity, thermophily and monophyly from environmental rRNA sequences. Proc Natl Acad Sci U S A 93:9188–9193. doi:10.1073/pnas.93.17.9188. 8799176 PMC38617

[2] Inagaki F, Suzuki M, Takai K, Oida H, Sakamoto T, Aoki K, Nealson KH, Horikoshi K. 2003. Microbial communities associated with geological horizons in coastal subseafloor sediments from the Sea of Okhotsk. Appl Environ Microbiol 69:7224–7235. doi:10.1128/AEM.69.12.7224-7235.2003. 14660370 PMC309994

[3] Lloyd KG, Schreiber L, Petersen DG, Kjeldsen KU, Lever MA, Steen AD, Stepanauskas R, Richter M, Kleindienst S, Lenk S, Schramm A, Jørgensen BB. 2013. Predominant archaea in marine sediments degrade detrital proteins. Nature 496:215–218. doi:10.1038/nature12033. 23535597

[4] Meng J, Xu J, Qin D, He Y, Xiao X, Wang F. 2014. Genetic and functional properties of uncultivated MCG archaea assessed by metagenome and gene expression analyses. ISME J 8:650–659. doi:10.1038/ismej.2013.174. 24108328 PMC3930316

[5] Jiang H, Dong H, Zhang G, Yu B, Chapman LR, Fields MW. 2006. Microbial diversity in water and sediment of Lake Chaka, an athalassohaline lake in northwestern China. Appl Environ Microbiol 72:3832–3845. doi:10.1128/AEM.02869-05. 16751487 PMC1489620

[6] Lloyd KG, Lapham L, Teske A. 2006. An anaerobic methane-oxidizing community of ANME-1b Archaea in hypersaline Gulf of Mexico sediments. Appl Environ Microbiol 72:7218–7230. doi:10.1128/AEM.00886-06. 16980428 PMC1636178

[7] Teske A, Sørensen KB. 2008. Uncultured Archaea in deep marine subsurface sediments: have we caught them all? ISME J 2:3–18. doi:10.1038/ismej.2007.90. 18180743

[8] Auguet J-C, Triadó-Margarit X, Nomokonova N, Camarero L, Casamayor EO. 2012. Vertical segregation and phylogenetic characterization of ammonia-oxidizing Archaea in a deep oligotrophic lake. ISME J 6:1786–1797. doi:10.1038/ismej.2012.33. 22495069 PMC3425235

[9] Fry JC, Parkes RJ, Cragg BA, Weightman AJ, Webster G. 2008. Prokaryotic biodiversity and activity in the deep subseafloor biosphere. FEMS Microbiol Ecol 66:181–196. doi:10.1111/j.1574-6941.2008.00566.x. 18752622

[10] Parkes RJ, Webster G, Cragg BA, Weightman AJ, Newberry CJ, Ferdelman TG, Kallmeyer J, Jørgensen BB, Aiello IW, Fry JC. 2005. Deep sub-seafloor prokaryotes stimulated at interfaces over geological time. Nature 436:390–394. doi:10.1038/nature03796. 16034418

[11] Biddle JF, Lipp JS, Lever MA, Lloyd KG, Sørensen KB, Anderson R, Fredricks HF, Elvert M, Kelly TJ, Schrag DP, Sogin ML, Brenchley JE, Teske A, House CH, Hinrichs K-U. 2006. Heterotrophic Archaea dominate sedimentary subsurface ecosystems off Peru. Proc Natl Acad Sci U S A 103:3846–3851. doi:10.1073/pnas.0600035103. 16505362 PMC1533785

[12] Kubo K, Lloyd KG, Biddle JF, Amann R, Teske A, Knittel K. 2012. Archaea of the Miscellaneous Crenarchaeotal Group are abundant, diverse and widespread in marine sediments. ISME J 6:1949–1965. doi:10.1038/ismej.2012.37. 22551871 PMC3449235

[13] Rinke C, Chuvochina M, Mussig AJ, Chaumeil P-A, Waite DW, Whitman WB, Parks DH, Hugenholtz P. 2021. A rank-normalized archaeal taxonomy based on genome phylogeny resolves widespread incomplete and uneven classifications. Nat Microbiol doi:10.1101/2020.03.01.972265.

[14] Fillol M, Auguet J-C, Casamayor EO, Borrego CM. 2016. Insights in the ecology and evolutionary history of the Miscellaneous Crenarchaeotic Group lineage. ISME J 10:665–677. doi:10.1038/ismej.2015.143. 26284443 PMC4817671

[15] Lazar CS, Biddle JF, Meador TB, Blair N, Hinrichs K-U, Teske AP. 2015. Environmental controls on intragroup diversity of the uncultured benthic archaea of the miscellaneous Crenarchaeotal group lineage naturally enriched in anoxic sediments of the White Oak River estuary (North Carolina, USA). Environ Microbiol 17:2228–2238. doi:10.1111/1462-2920.12659. 25331558

[16] Zhou Z, Pan J, Wang F, Gu J-D, Li M. 2018. Bathyarchaeota: globally distributed metabolic generalists in anoxic environments. FEMS Microbiol Rev 42:639–655. doi:10.1093/femsre/fuy023. 29790926

[17] He Y, Li M, Perumal V, Feng X, Fang J, Xie J, Sievert SM, Wang F. 2016. Genomic and enzymatic evidence for acetogenesis among multiple lineages of the archaeal phylum Bathyarchaeota widespread in marine sediments. Nat Microbiol 1:16035. doi:10.1038/nmicrobiol.2016.35. 27572832

[18] Lazar CS, Baker BJ, Seitz K, Hyde AS, Dick GJ, Hinrichs K-U, Teske AP. 2016. Genomic evidence for distinct carbon substrate preferences and ecological niches of Bathyarchaeota in estuarine sediments. Environ Microbiol 18:1200–1211. doi:10.1111/1462-2920.13142. 26626228

[19] Zhang W, Ding W, Yang B, Tian R, Gu S, Luo H, Qian P-Y. 2016. Genomic and transcriptomic evidence for carbohydrate consumption among microorganisms in a cold seep brine pool. Front Microbiol 7:1825. doi:10.3389/fmicb.2016.01825. 27895636 PMC5108811

[20] Seyler LM, McGuinness LM, Kerkhof LJ. 2014. Crenarchaeal heterotrophy in salt marsh sediments. ISME J 8:1534–1543. doi:10.1038/ismej.2014.15. 24553469 PMC4069397

[21] Yu T, Wu W, Liang W, Lever MA, Hinrichs K-U, Wang F. 2018. Growth of sedimentary Bathyarchaeota on lignin as an energy source. Proc Natl Acad Sci U S A 115:6022–6027. doi:10.1073/pnas.1718854115. 29773709 PMC6003339

[22] Feng X, Wang Y, Zubin R, Wang F. 2019. Core metabolic features and hot origin of Bathyarchaeota. Engineering 5:498–504. doi:10.1016/j.eng.2019.01.011.

[23] Evans PN, Parks DH, Chadwick GL, Robbins SJ, Orphan VJ, Golding SD, Tyson GW. 2015. Methane metabolism in the archaeal phylum Bathyarchaeota revealed by genome-centric metagenomics. Science 350:434–438. doi:10.1126/science.aac7745. 26494757

[24] Vanwonterghem I, Evans PN, Parks DH, Jensen PD, Woodcroft BJ, Hugenholtz P, Tyson GW. 2016. Methylotrophic methanogenesis discovered in the archaeal phylum Verstraetearchaeota. Nat Microbiol 1:16170. doi:10.1038/nmicrobiol.2016.170. 27694807

[25] Laso-Pérez R, Wegener G, Knittel K, Widdel F, Harding KJ, Krukenberg V, Meier DV, Richter M, Tegetmeyer HE, Riedel D, Richnow H-H, Adrian L, Reemtsma T, Lechtenfeld OJ, Musat F. 2016. Thermophilic archaea activate butane via alkyl-coenzyme M formation. Nature 539:396–401. doi:10.1038/nature20152. 27749816

[26] Evans PN, Boyd JA, Leu AO, Woodcroft BJ, Parks DH, Hugenholtz P, Tyson GW. 2019. An evolving view of methane metabolism in the Archaea. Nat Rev Microbiol 17:219–232. doi:10.1038/s41579-018-0136-7. 30664670

[27] Hua Z-S, Wang Y-L, Evans PN, Qu Y-N, Goh KM, Rao Y-Z, Qi Y-L, Li Y-X, Huang M-J, Jiao J-Y, Chen Y-T, Mao Y-P, Shu W-S, Hozzein W, Hedlund BP, Tyson GW, Zhang T, Li W-J. 2019. Insights into the ecological roles and evolution of methyl-coenzyme M reductase-containing hot spring Archaea. Nat Commun 10:4574. doi:10.1038/s41467-019-12574-y. 31594929 PMC6783470

[28] Parks DH, Chuvochina M, Chaumeil P-A, Rinke C, Mussig AJ, Hugenholtz P. 2019. Selection of representative genomes for 24,706 bacterial and archaeal species clusters provide a complete genome-based taxonomy. bioRxiv doi:10.1101/771964.

[29] Bowers RM, Kyrpides NC, Stepanauskas R, Harmon-Smith M, Doud D, Reddy TBK, Schulz F, Jarett J, Rivers AR, Eloe-Fadrosh EA, Tringe SG, Ivanova NN, Copeland A, Clum A, Becraft ED, Malmstrom RR, Birren B, Podar M, Bork P, Weinstock GM, Garrity GM, Dodsworth JA, Yooseph S, Sutton G, Glöckner FO, Gilbert JA, Nelson WC, Hallam SJ, Jungbluth SP, Ettema TJG, Tighe S, Konstantinidis KT, Liu W-T, Baker BJ, Rattei T, Eisen JA, Hedlund B, McMahon KD, Fierer N, Knight R, Finn R, Cochrane G, Karsch-Mizrachi I, Tyson GW, Rinke C, Lapidus A, Meyer F, Yilmaz P, Parks DH, Eren AM, Schriml L, Banfield JF, Genome Standards Consortium, et al. 2017. Minimum information about a single amplified genome (MISAG) and a metagenome-assembled genome (MIMAG) of bacteria and archaea. Nat Biotechnol 35:725–731. doi:10.1038/nbt.3893. 28787424 PMC6436528

[30] Probst AJ, Ladd B, Jarett JK, Geller-McGrath DE, Sieber CMK, Emerson JB, Anantharaman K, Thomas BC, Malmstrom RR, Stieglmeier M, Klingl A, Woyke T, Ryan MC, Banfield JF. 2018. Differential depth distribution of microbial function and putative symbionts through sediment-hosted aquifers in the deep terrestrial subsurface. Nat Microbiol 3:328–336. doi:10.1038/s41564-017-0098-y. 29379208 PMC6792436

[31] Anantharaman K, Brown CT, Hug LA, Sharon I, Castelle CJ, Probst AJ, Thomas BC, Singh A, Wilkins MJ, Karaoz U, Brodie EL, Williams KH, Hubbard SS, Banfield JF. 2016. Thousands of microbial genomes shed light on interconnected biogeochemical processes in an aquifer system. Nat Commun 7:13219. doi:10.1038/ncomms13219. 27774985 PMC5079060

[32] Parks DH, Rinke C, Chuvochina M, Chaumeil P-A, Woodcroft BJ, Evans PN, Hugenholtz P, Tyson GW. 2017. Recovery of nearly 8,000 metagenome-assembled genomes substantially expands the tree of life. Nat Microbiol 2:1533–1542. doi:10.1038/s41564-017-0012-7. 28894102

[33] Dombrowski N, Seitz KW, Teske AP, Baker BJ. 2017. Genomic insights into potential interdependencies in microbial hydrocarbon and nutrient cycling in hydrothermal sediments. Microbiome 5:106. doi:10.1186/s40168-017-0322-2. 28835260 PMC5569505

[34] Jungbluth SP, Amend JP, Rappé MS. 2017. Metagenome sequencing and 98 microbial genomes from Juan de Fuca Ridge flank subsurface fluids. Sci Data 4:170037. doi:10.1038/sdata.2017.37. 28350381 PMC5369317

[35] Dong X, Greening C, Rattray JE, Chakraborty A, Chuvochina M, Mayumi D, Dolfing J, Li C, Brooks JM, Bernard BB, Groves RA, Lewis IA, Hubert CRJ. 2019. Metabolic potential of uncultured bacteria and archaea associated with petroleum seepage in deep-sea sediments. Nat Commun 10:1816. doi:10.1038/s41467-019-09747-0. 31000700 PMC6472368

[36] Tully BJ, Graham ED, Heidelberg JF. 2018. The reconstruction of 2,631 draft metagenome-assembled genomes from the global oceans. Sci Data 5:170203. doi:10.1038/sdata.2017.203. 29337314 PMC5769542

[37] Butterfield CN, Li Z, Andeer PF, Spaulding S, Thomas BC, Singh A, Hettich RL, Suttle KB, Probst AJ, Tringe SG, Northen T, Pan C, Banfield JF. 2016. Proteogenomic analyses indicate bacterial methylotrophy and archaeal heterotrophy are prevalent below the grass root zone. PeerJ 4:e2687. doi:10.7717/peerj.2687. 27843720 PMC5103831

[38] Parks DH, Chuvochina M, Waite DW, Rinke C, Skarshewski A, Chaumeil P-A, Hugenholtz P. 2018. A standardized bacterial taxonomy based on genome phylogeny substantially revises the tree of life. Nat Biotechnol 36:996–1004. doi:10.1038/nbt.4229. 30148503

[39] Goenrich M, Thauer RK, Yurimoto H, Kato N. 2005. Formaldehyde activating enzyme (Fae) and hexulose-6-phosphate synthase (Hps) in Methanosarcina barkeri: a possible function in ribose-5-phosphate biosynthesis. Arch Microbiol 184:41–48. doi:10.1007/s00203-005-0008-1. 16075199

[40] Blumer-Schuette SE, Kataeva I, Westpheling J, Adams MW, Kelly RM. 2008. Extremely thermophilic microorganisms for biomass conversion: status and prospects. Curr Opin Biotechnol 19:210–217. doi:10.1016/j.copbio.2008.04.007. 18524567

[41] Webster G, Rinna J, Roussel EG, Fry JC, Weightman AJ, Parkes RJ. 2010. Prokaryotic functional diversity in different biogeochemical depth zones in tidal sediments of the Severn Estuary, UK, revealed by stable-isotope probing. FEMS Microbiol Ecol 72:179–197. doi:10.1111/j.1574-6941.2010.00848.x. 20337706

[42] Meador TB, Bowles M, Lazar CS, Zhu C, Teske A, Hinrichs K-U. 2015. The archaeal lipidome in estuarine sediment dominated by members of the Miscellaneous Crenarchaeotal Group. Environ Microbiol 17:2441–2458. doi:10.1111/1462-2920.12716. 25403417

[43] Na H, Lever MA, Kjeldsen KU, Schulz F, Jørgensen BB. 2015. Uncultured Desulfobacteraceae and Crenarchaeotal group C3 incorporate 13C-acetate in coastal marine sediment. Environ Microbiol Rep 7:614–622. doi:10.1111/1758-2229.12296. 25950866

[44] Adam PS, Borrel G, Gribaldo S. 2019. An archaeal origin of the Wood-Ljungdahl H 4 MPT branch and the emergence of bacterial methylotrophy. Nat Microbiol 4:2155–2163. doi:10.1038/s41564-019-0534-2. 31451772

[45] Sato T, Atomi H, Imanaka T. 2007. Archaeal type III RuBisCOs function in a pathway for AMP metabolism. Science 315:1003–1006. doi:10.1126/science.1135999. 17303759

[46] Sabath N, Ferrada E, Barve A, Wagner A. 2013. Growth temperature and genome size in bacteria are negatively correlated, suggesting genomic streamlining during thermal adaptation. Genome Biol Evol 5:966–977. doi:10.1093/gbe/evt050. 23563968 PMC3673621

[47] Mira A, Ochman H, Moran NA. 2001. Deletional bias and the evolution of bacterial genomes. Trends Genet 17:589–596. doi:10.1016/s0168-9525(01)02447-7. 11585665

[48] Kuo C-H, Moran NA, Ochman H. 2009. The consequences of genetic drift for bacterial genome complexity. Genome Res 19:1450–1454. doi:10.1101/gr.091785.109. 19502381 PMC2720180

[49] Richter K, Haslbeck M, Buchner J. 2010. The heat shock response: life on the verge of death. Mol Cell 40:253–266. doi:10.1016/j.molcel.2010.10.006. 20965420

[50] Petitjean C, Moreira D, López-García P, Brochier-Armanet C. 2012. Horizontal gene transfer of a chloroplast DnaJ-Fer protein to Thaumarchaeota and the evolutionary history of the DnaK chaperone system in Archaea. BMC Evol Biol 12:226. doi:10.1186/1471-2148-12-226. 23181628 PMC3564930

[51] Boyd JA, Jungbluth SP, Leu AO, Evans PN, Woodcroft BJ, Chadwick GL, Orphan VJ, Amend JP, Rappé MS, Tyson GW. 2019. Divergent methyl-coenzyme M reductase genes in a deep-subseafloor Archaeoglobi. ISME J 13:1269–1279. doi:10.1038/s41396-018-0343-2. 30651609 PMC6474303

[52] Borrel G, Adam PS, McKay LJ, Chen L-X, Sierra-García IN, Sieber CMK, Letourneur Q, Ghozlane A, Andersen GL, Li W-J, Hallam SJ, Muyzer G, de Oliveira VM, Inskeep WP, Banfield JF, Gribaldo S. 2019. Wide diversity of methane and short-chain alkane metabolisms in uncultured archaea. Nat Microbiol 4:603–613. doi:10.1038/s41564-019-0363-3. 30833729 PMC6453112

[53] Wang Y, Wegener G, Hou J, Wang F, Xiao X. 2019. Expanding anaerobic alkane metabolism in the domain of Archaea. Nat Microbiol 4:595–602. doi:10.1038/s41564-019-0364-2. 30833728

[54] Seitz KW, Dombrowski N, Eme L, Spang A, Lombard J, Sieber JR, Teske AP, Ettema TJG, Baker BJ. 2019. Asgard archaea capable of anaerobic hydrocarbon cycling. Nat Commun 10:1822. doi:10.1038/s41467-019-09364-x. 31015394 PMC6478937

[55] Borrel G, Adam PS, Gribaldo S. 2016. Methanogenesis and the Wood-Ljungdahl pathway: an ancient, versatile, and fragile association. Genome Biol Evol 8:1706–1711. doi:10.1093/gbe/evw114. 27189979 PMC4943185

[56] Welte C, Deppenmeier U. 2011. Re-evaluation of the function of the F420 dehydrogenase in electron transport of Methanosarcina mazei. FEBS J 278:1277–1287. doi:10.1111/j.1742-4658.2011.08048.x. 21306561

[57] Lang K, Schuldes J, Klingl A, Poehlein A, Daniel R, Brune A. 2015. New mode of energy metabolism in the seventh order of methanogens as revealed by comparative genome analysis of “Candidatus Methanoplasma termitum”. Appl Environ Microbiol 81:1338–1352. doi:10.1128/AEM.03389-14. 25501486 PMC4309702

[58] Marreiros BC, Batista AP, Duarte AMS, Pereira MM. 2013. A missing link between complex I and group 4 membrane-bound [NiFe] hydrogenases. Biochim Biophys Acta 1827:198–209. doi:10.1016/j.bbabio.2012.09.012. 23000657

[59] Yan Z, Wang M, Ferry JG. 2017. A ferredoxin- and F420H2-dependent, electron-bifurcating, heterodisulfide reductase with homologs in the domains Bacteria and Archaea. mBio 8:e02285-16. doi:10.1128/mBio.02285-16. 28174314 PMC5296606

[60] Hua Z-S, Qu Y-N, Zhu Q, Zhou E-M, Qi Y-L, Yin Y-R, Rao Y-Z, Tian Y, Li Y-X, Liu L, Castelle CJ, Hedlund BP, Shu W-S, Knight R, Li W-J. 2018. Genomic inference of the metabolism and evolution of the archaeal phylum Aigarchaeota. Nat Commun 9:2832. doi:10.1038/s41467-018-05284-4. 30026532 PMC6053391

[61] Hua Z-S, Han Y-J, Chen L-X, Liu J, Hu M, Li S-J, Kuang J-L, Chain PS, Huang L-N, Shu W-S. 2015. Ecological roles of dominant and rare prokaryotes in acid mine drainage revealed by metagenomics and metatranscriptomics. ISME J 9:1280–1294. doi:10.1038/ismej.2014.212. 25361395 PMC4438317

[62] Bankevich A, Nurk S, Antipov D, Gurevich AA, Dvorkin M, Kulikov AS, Lesin VM, Nikolenko SI, Pham S, Prjibelski AD, Pyshkin AV, Sirotkin AV, Vyahhi N, Tesler G, Alekseyev MA, Pevzner PA. 2012. SPAdes: a new genome assembly algorithm and its applications to single-cell sequencing. J Comput Biol 19:455–477. doi:10.1089/cmb.2012.0021. 22506599 PMC3342519

[63] Kang DD, Froula J, Egan R, Wang Z. 2015. MetaBAT, an efficient tool for accurately reconstructing single genomes from complex microbial communities. PeerJ 3:e1165. doi:10.7717/peerj.1165. 26336640 PMC4556158

[64] Dick GJ, Andersson AF, Baker BJ, Simmons SL, Thomas BC, Yelton AP, Banfield JF. 2009. Community-wide analysis of microbial genome sequence signatures. Genome Biol 10:R85. doi:10.1186/gb-2009-10-8-r85. 19698104 PMC2745766

[65] Parks DH, Imelfort M, Skennerton CT, Hugenholtz P, Tyson GW. 2015. CheckM: assessing the quality of microbial genomes recovered from isolates, single cells, and metagenomes. Genome Res 25:1043–1055. doi:10.1101/gr.186072.114. 25977477 PMC4484387

[66] Hyatt D, Chen G-L, LoCascio PF, Land ML, Larimer FW, Hauser LJ. 2010. Prodigal: prokaryotic gene recognition and translation initiation site identification. BMC Bioinformatics 11:119. doi:10.1186/1471-2105-11-119. 20211023 PMC2848648

[67] Kanehisa M, Furumichi M, Tanabe M, Sato Y, Morishima K. 2017. KEGG: new perspectives on genomes, pathways, diseases and drugs. Nucleic Acids Res 45:D353–D361. doi:10.1093/nar/gkw1092. 27899662 PMC5210567

[68] Makarova KS, Wolf YI, Koonin EV. 2015. Archaeal Clusters of Orthologous Genes (arCOGs): an update and application for analysis of shared features between Thermococcales, Methanococcales, and Methanobacteriales. Life (Basel) 5:818–840. doi:10.3390/life5010818. 25764277 PMC4390880

[69] Finn RD, Coggill P, Eberhardt RY, Eddy SR, Mistry J, Mitchell AL, Potter SC, Punta M, Qureshi M, Sangrador-Vegas A, Salazar GA, Tate J, Bateman A. 2016. The Pfam protein families database: towards a more sustainable future. Nucleic Acids Res 44:D279–D285. doi:10.1093/nar/gkv1344. 26673716 PMC4702930

[70] Buchfink B, Xie C, Huson DH. 2015. Fast and sensitive protein alignment using DIAMOND. Nat Methods 12:59–60. doi:10.1038/nmeth.3176. 25402007

[71] Zhang H, Yohe T, Huang L, Entwistle S, Wu P, Yang Z, Busk PK, Xu Y, Yin Y. 2018. dbCAN2: a meta server for automated carbohydrate-active enzyme annotation. Nucleic Acids Res 46:W95–W101. doi:10.1093/nar/gky418. 29771380 PMC6031026

[72] Lombard V, Golaconda Ramulu H, Drula E, Coutinho PM, Henrissat B. 2014. The carbohydrate-active enzymes database (CAZy) in 2013. Nucleic Acids Res 42:D490–D495. doi:10.1093/nar/gkt1178. 24270786 PMC3965031

[73] CAZypedia Consortium. 2018. Ten years of CAZypedia: a living encyclopedia of carbohydrate-active enzymes. Glycobiology 28:3–8. doi:10.1093/glycob/cwx089. 29040563

[74] Stam MR, Danchin EGJ, Rancurel C, Coutinho PM, Henrissat B. 2006. Dividing the large glycoside hydrolase family 13 into subfamilies: towards improved functional annotations of α-amylase-related proteins. Protein Eng Des Sel 19:555–562. doi:10.1093/protein/gzl044. 17085431

[75] van den Brink J, de Vries RP. 2011. Fungal enzyme sets for plant polysaccharide degradation. Appl Microbiol Biotechnol 91:1477–1492. doi:10.1007/s00253-011-3473-2. 21785931 PMC3160556

[76] Aspeborg H, Coutinho PM, Wang Y, Brumer H, Henrissat B. 2012. Evolution, substrate specificity and subfamily classification of glycoside hydrolase family 5 (GH5). BMC Evol Biol 12:186. doi:10.1186/1471-2148-12-186. 22992189 PMC3526467

[77] Baker BJ, Lazar CS, Teske AP, Dick GJ. 2015. Genomic resolution of linkages in carbon, nitrogen, and sulfur cycling among widespread estuary sediment bacteria. Microbiome 3:14. doi:10.1186/s40168-015-0077-6. 25922666 PMC4411801

[78] Chang H-X, Yendrek CR, Caetano-Anolles G, Hartman GL. 2016. Genomic characterization of plant cell wall degrading enzymes and in silico analysis of xylanses and polygalacturonases of Fusarium virguliforme. BMC Microbiol 16. doi:10.1186/s12866-016-0761-0. PMC4941037 27405320

[79] Chuzel L, Ganatra MB, Rapp E, Henrissat B, Taron CH. 2018. Functional metagenomics identifies an exosialidase with an inverting catalytic mechanism that defines a new glycoside hydrolase family (GH156). J Biol Chem 293:18138–18150. doi:10.1074/jbc.RA118.003302. 30249617 PMC6254351

[80] Nguyen STC, Freund HL, Kasanjian J, Berlemont R. 2018. Function, distribution, and annotation of characterized cellulases, xylanases, and chitinases from CAZy. Appl Microbiol Biotechnol 102:1629–1637. doi:10.1007/s00253-018-8778-y. 29359269 PMC5806127

[81] Liu N, Li H, Chevrette MG, Zhang L, Cao L, Zhou H, Zhou X, Zhou Z, Pope PB, Currie CR, Huang Y, Wang Q. 2019. Functional metagenomics reveals abundant polysaccharide-degrading gene clusters and cellobiose utilization pathways within gut microbiota of a wood-feeding higher termite. ISME J 13:104–117. doi:10.1038/s41396-018-0255-1. 30116044 PMC6298952

[82] Chaumeil P-A, Mussig AJ, Hugenholtz P, Parks DH. 2019. GTDB-Tk: a toolkit to classify genomes with the Genome Taxonomy Database. Bioinformatics 36:1925–1927. doi:10.1093/bioinformatics/btz848. PMC7703759 31730192

[83] Nguyen L-T, Schmidt HA, von Haeseler A, Minh BQ. 2015. IQ-TREE: a fast and effective stochastic algorithm for estimating maximum-likelihood phylogenies. Mol Biol Evol 32:268–274. doi:10.1093/molbev/msu300. 25371430 PMC4271533

[84] Kalyaanamoorthy S, Minh BQ, Wong TKF, von Haeseler A, Jermiin LS. 2017. ModelFinder: fast model selection for accurate phylogenetic estimates. Nat Methods 14:587–589. doi:10.1038/nmeth.4285. 28481363 PMC5453245

[85] Lagesen K, Hallin P, Rødland EA, Staerfeldt H-H, Rognes T, Ussery DW. 2007. RNAmmer: consistent and rapid annotation of ribosomal RNA genes. Nucleic Acids Res 35:3100–3108. doi:10.1093/nar/gkm160. 17452365 PMC1888812

[86] Morgulis A, Coulouris G, Raytselis Y, Madden TL, Agarwala R, Schäffer AA. 2008. Database indexing for production MegaBLAST searches. Bioinformatics 24:1757–1764. doi:10.1093/bioinformatics/btn322. 18567917 PMC2696921

[87] Cole JR, Wang Q, Fish JA, Chai B, McGarrell DM, Sun Y, Brown CT, Porras-Alfaro A, Kuske CR, Tiedje JM. 2014. Ribosomal Database Project: data and tools for high throughput rRNA analysis. Nucleic Acids Res 42:D633–D642. doi:10.1093/nar/gkt1244. 24288368 PMC3965039

[88] Katoh K, Misawa K, Kuma K, Miyata T. 2002. MAFFT: a novel method for rapid multiple sequence alignment based on fast Fourier transform. Nucleic Acids Res 30:3059–3066. doi:10.1093/nar/gkf436. 12136088 PMC135756

[89] Capella-Gutiérrez S, Silla-Martínez JM, Gabaldón T. 2009. trimAl: a tool for automated alignment trimming in large-scale phylogenetic analyses. Bioinformatics 25:1972–1973. doi:10.1093/bioinformatics/btp348. 19505945 PMC2712344

[90] Edgar RC. 2004. MUSCLE: multiple sequence alignment with high accuracy and high throughput. Nucleic Acids Res 32:1792–1797. doi:10.1093/nar/gkh340. 15034147 PMC390337

[91] Letunic I, Bork P. 2016. Interactive tree of life (iTOL) v3: an online tool for the display and annotation of phylogenetic and other trees. Nucleic Acids Res 44:W242–W245. doi:10.1093/nar/gkw290. 27095192 PMC4987883

[92] Oksanen J, Blanchet FG, Friendly M, Kindt R, Legendre P, McGlinn D, Minchin PR, O’Hara RB, Simpson GL, Solymos P, Stevens MHH, Szoecs E, Wagner H. 2020. vegan: Community Ecology Package.

[93] Szöllõsi GJ, Rosikiewicz W, Boussau B, Tannier E, Daubin V. 2013. Efficient exploration of the space of reconciled gene trees. Syst Biol 62:901–912. doi:10.1093/sysbio/syt054. 23925510 PMC3797637

