# Supplementary figures and images for: Comparative Genomics Reveals Thermal Adaptation and a High Metabolic Diversity in “Candidatus Bathyarchaeia”
Source: mSystems. 2021 Jul 20;6(4):e00252-21. doi: 10.1128/mSystems.00252-21 (PMC8407382; doi:10.1128/mSystems.00252-21)

Supplementary Figures

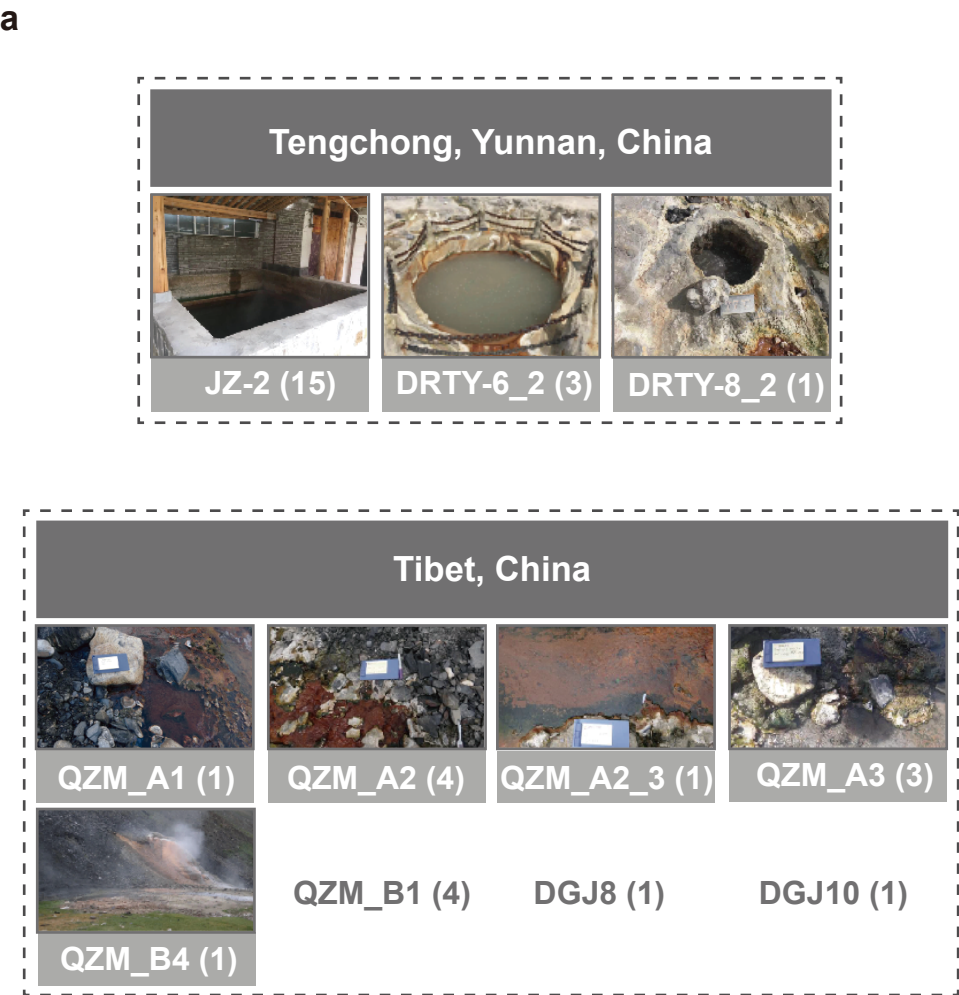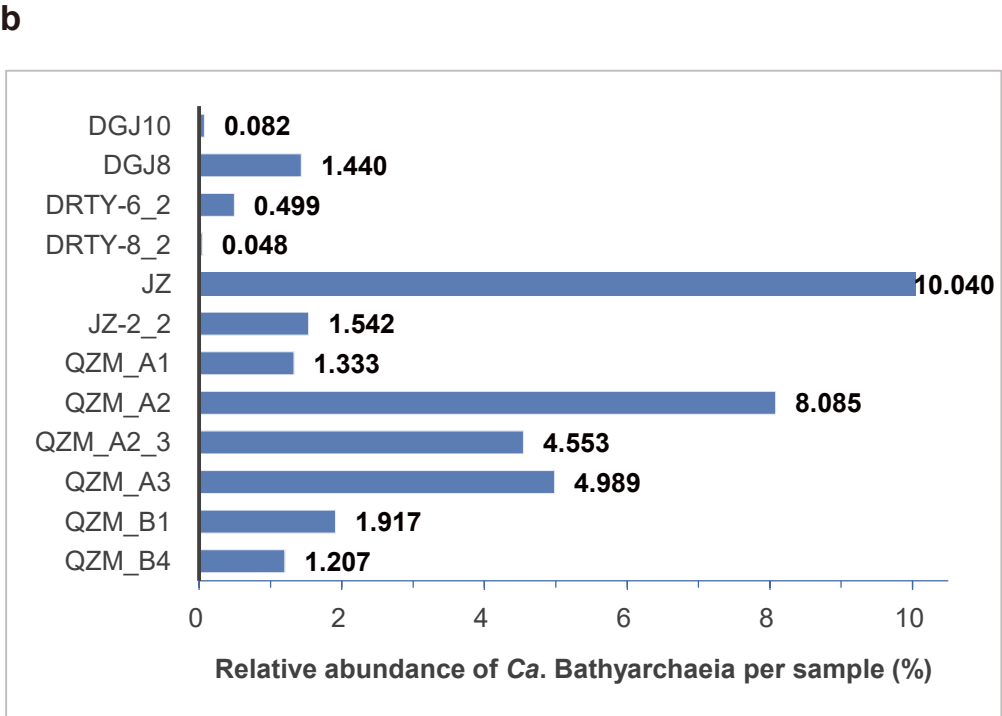

Supplement: FIG S1 [file msystems.00252-21-sf001.pdf]

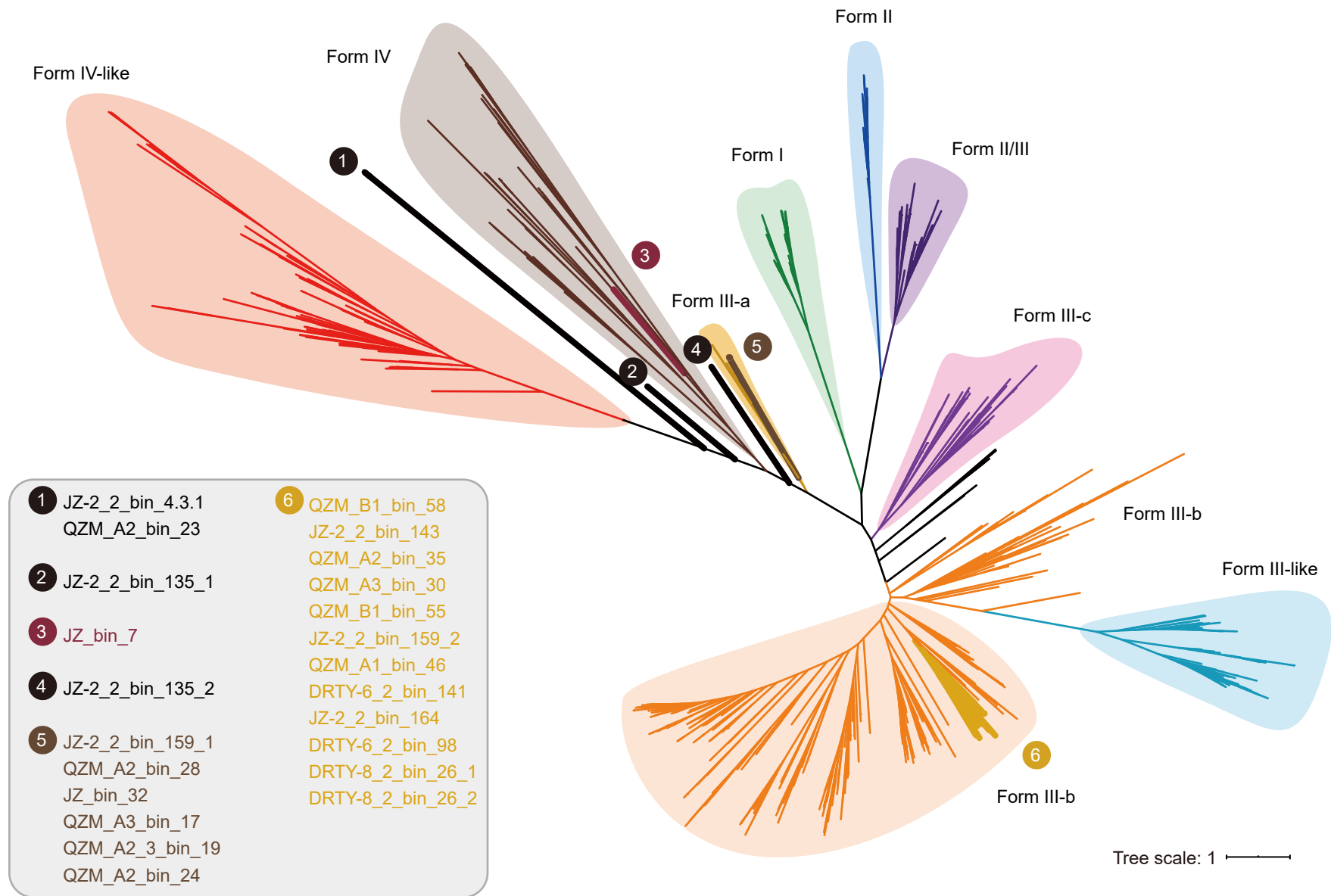

Supplement: FIG S2 [file msystems.00252-21-sf002.pdf]

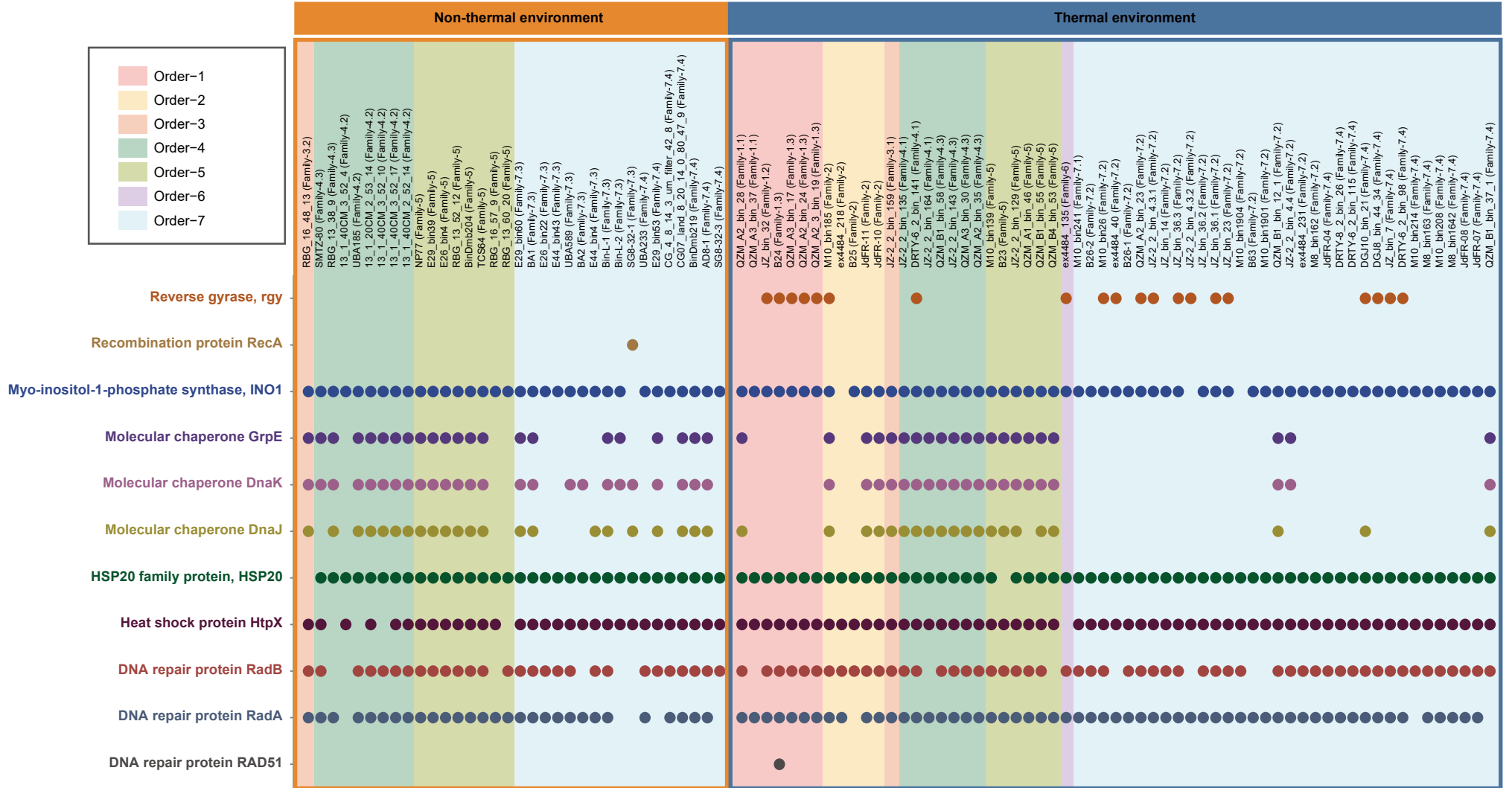

Supplement: FIG S3 [file msystems.00252-21-sf003.pdf]

Tree scale: 0.1

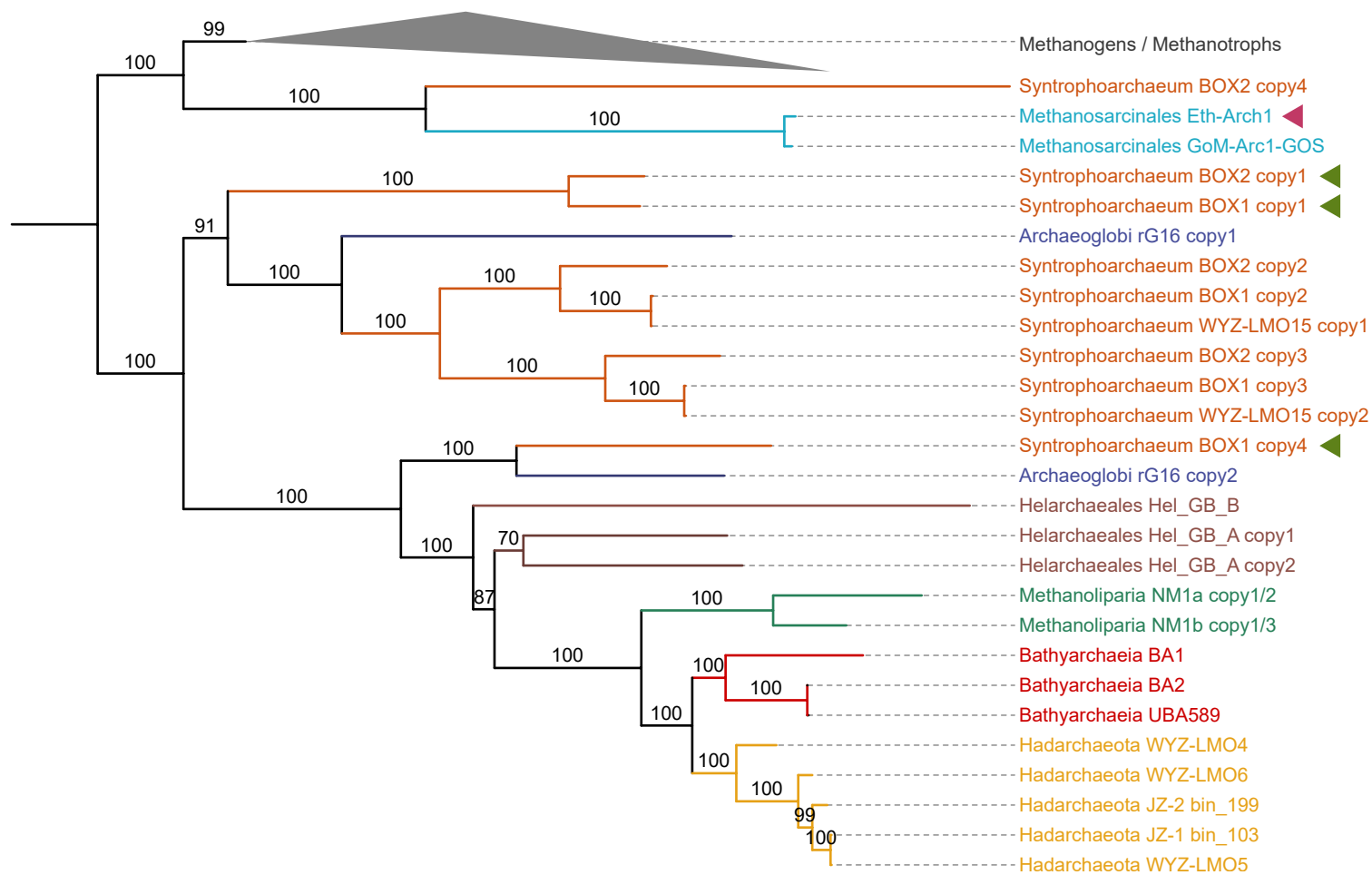

Alkanotrophs

Ethane oxidation  
 Butane and/or propane oxidation

Supplement: FIG S4 [file msystems.00252-21-sf004.pdf]
