# Supplementary material for: Comparative Genomics Reveals Thermal Adaptation and a High Metabolic Diversity in “Candidatus Bathyarchaeia”
Source: mSystems. 2021 Jul 20;6(4):e00252-21. doi: 10.1128/mSystems.00252-21 (PMC8407382; doi:10.1128/mSystems.00252-21)
Supplement: TABLE S1 [file msystems.00252-21-st001.pdf]

Supplementary Table 1. Overview of 35 *Ca* . Bathyarchaeia MAGs from hot springs.

Order	Family	Genome bins	No. of scaffolds	Genome size (Mb)	GC content (%)	N50 value (bp)	Relative abundance of genome bins (%)	No. of protein coding genes	Coding density (%)	No. of genes annotated by KO	No. of genes annotated by COG	No. of genes annotated by arCOG	No. of genes annotated by pfam	Completeness‡	Contamination‡	Heterogeneity‡
Order-1	Family-1.1	QZM_A2_bin_28	143	1.67	47.28	14757	0.44	1724	79.9	814(47.2%)	1165(67.6%)	1182(68.6%)	1199(69.5%)	85.60	1.94	0
		QZM_A3_bin_37	181	1.11	46.70	6560	0.23	1184	76.8	572(48.3%)	805(68.0%)	809(68.3%)	824(69.6%)	68.59	1.25	0
	Family-1.2	JZ_bin_32	14	1.41	41.04	176051	0.71	1442	91.0	816(56.6%)	1085(75.2%)	1095(75.9%)	1130(78.4%)	93.93	0.93	0
	Family-1.3	QZM_A2_3_bin_19	194	1.30	40.61	7558	0.58	1526	85.0	680(44.6%)	996(65.3%)	991(64.9%)	1024(67.1%)	74.68	0.97	0
		QZM_A2_bin_24	74	1.98	41.39	72377	1.91	2113	86.8	930(44.0%)	1399(66.2%)	1416(67.0%)	1491(70.6%)	98.06	2.43	0
		QZM_A3_bin_17	129	1.90	41.30	24870	0.62	2085	86.1	903(43.3%)	1350(64.7%)	1365(65.5%)	1419(68.1%)	88.50	0	0
Order-3	Family-3.1	JZ-2_2_bin_159	30	1.39	42.81	88026	0.06	1496	87.7	814(54.4%)	1105(73.9%)	1133(75.7%)	1170(78.2%)	98.13	0.47	0
Order-4	Family-4.1	DRTY-6_2_bin_141	12	1.49	30.94	354801	0.08	1564	88.0	854(54.6%)	1171(74.9%)	1206(77.1%)	1215(77.7%)	98.13	0.93	0
		JZ-2_2_bin_135	32	1.75	52.83	119633	0.11	1826	84.6	919(50.3%)	1302(71.3%)	1343(73.5%)	1391(76.2%)	96.26	0.93	0
		JZ-2_2_bin_164	24	1.33	32.45	90957	0.08	1439	88.8	811(56.4%)	1085(75.4%)	1133(78.7%)	1128(78.4%)	97.20	0	0
	Family-4.3	JZ-2_2_bin_143	42	1.63	45.49	48958	0.06	1806	87.7	911(50.4%)	1287(71.3%)	1329(73.6%)	1368(75.7%)	97.20	0	0
		QZM_A2_bin_35	132	1.57	45.89	16787	0.35	1872	86.4	869(46.4%)	1246(66.6%)	1302(69.6%)	1307(69.8%)	81.55	0	0
		QZM_A3_bin_30	187	1.31	45.39	7962	0.27	1610	83.9	726(45.1%)	1036(64.3%)	1072(66.6%)	1078(67.0%)	69.54	0	0
		QZM_B1_bin_58	156	1.70	51.81	15081	0.33	1991	84.7	878(44.1%)	1245(62.5%)	1263(63.4%)	1338(67.2%)	75.24	0.97	0
Order-5	Family-5	JZ-2_2_bin_129	43	1.83	50.84	99040	0.33	2000	85.8	901(45.1%)	1343(67.2%)	1375(68.8%)	1419(71.0%)	94.86	0	0
		QZM_A1_bin_46	162	1.42	52.05	11161	0.27	1625	84.1	761(46.8%)	1110(68.3%)	1129(69.5%)	1161(71.4%)	62.56	0	0
		QZM_B1_bin_55	91	1.90	43.28	51168	0.65	2117	86.6	1014(47.9%)	1442(68.1%)	1445(68.3%)	1508(71.2%)	92.99	0.93	0
		QZM_B4_bin_53	122	1.32	43.16	15217	0.30	1517	87.2	776(51.2%)	1081(71.3%)	1086(71.6%)	1142(75.3%)	82.24	0.93	0
Order-7	Family-7.2	JZ_bin_14	75	1.10	45.89	24211	1.97	1197	91.1	695(58.1%)	928(77.5%)	941(78.6%)	965(80.6%)	88.32	0.93	0
		JZ_bin_23	42	1.46	43.61	78088	1.80	1558	89.8	878(56.4%)	1215(78.0%)	1241(79.7%)	1225(78.6%)	99.07	4.21	0
		JZ_bin_36.1	108	1.49	42.65	20585	0.54	1584	89.6	848(53.5%)	1185(74.8%)	1216(76.8%)	1200(75.8%)	95.79	2.80	0
		JZ_bin_36.2	35	1.45	44.45	89595	0.80	1503	90.9	835(55.6%)	1168(77.7%)	1180(78.5%)	1220(81.2%)	98.13	4.67	0
		JZ_bin_36.3	48	1.32	43.44	38869	0.85	1388	90.5	753(54.3%)	1040(74.9%)	1055(76.0%)	1062(76.5%)	93.46	1.87	0
		JZ-2_2_bin_4.3.1	91	1.39	42.76	21007	0.06	1470	88.6	758(51.6%)	1093(74.4%)	1082(73.6%)	1123(76.4%)	89.32	0.97	0
		JZ-2_2_bin_4.3.2	262	1.57	41.23	6926	0.05	1834	87.7	835(45.5%)	1228(67.0%)	1235(67.3%)	1290(70.3%)	74.45	0.31	0
		JZ-2_2_bin_4.4	46	1.20	41.41	52385	0.10	1252	87.4	619(49.4%)	875(69.9%)	875(69.9%)	945(75.5%)	78.43	0	0
		QZM_A2_bin_23	120	1.74	42.81	20498	0.50	1907	86.4	893(46.8%)	1295(67.9%)	1312(68.8%)	1354(71.0%)	91.92	0.97	0
		QZM_B1_bin_12_1	92	1.41	42.29	19348	0.32	1533	87.5	707(46.1%)	1021(66.6%)	1030(67.2%)	1081(70.5%)	87.85	0	0
	Family-7.4	DGJ10_bin_21	358	1.26	43.35	4821	0.07	1653	86.0	818(49.5%)	1084(65.6%)	1109(67.1%)	1102(66.7%)	75.40	0	0
		DGJ8_bin_44_34	83	1.37	43.93	21477	1.01	1639	87.7	806(49.2%)	1088(66.4%)	1116(68.1%)	1122(68.5%)	83.18	1.87	0
		DRTY-6_2_bin_115	15	1.31	42.86	187909	0.20	1441	91.6	704(48.9%)	1000(69.4%)	1008(70.0%)	1042(72.3%)	88.79	1.87	0
		DRTY-6_2_bin_98	9	1.71	43.23	240134	0.15	1837	90.2	918(50.0%)	1292(70.3%)	1332(72.5%)	1349(73.4%)	99.07	2.80	0
		DRTY-8_2_bin_26	155	1.08	41.57	8502	0.03	1331	89.7	769(57.8%)	991(74.5%)	1024(76.9%)	1001(75.2%)	87.85	1.87	0
		JZ_bin_7	41	1.24	45.89	37224	3.09	1394	92.6	767(55.0%)	1034(74.2%)	1047(75.1%)	1071(76.8%)	94.93	0.93	0
		QZM_B1_bin_37_1	126	1.64	45.01	21240	0.32	1929	88.9	810(42.0%)	1154(59.8%)	1189(61.6%)	1257(65.2%)	90.20	0.93	0

‡ Completeness, contamination and heterogeneity are estimated by CheckM (Parks et al., 2015).
