# Supplementary material for: Comparative Genomics Reveals Thermal Adaptation and a High Metabolic Diversity in “Candidatus Bathyarchaeia”
Source: mSystems. 2021 Jul 20;6(4):e00252-21. doi: 10.1128/mSystems.00252-21 (PMC8407382; doi:10.1128/mSystems.00252-21)
Supplement: FIG S5 [file msystems.00252-21-sf005.pdf]

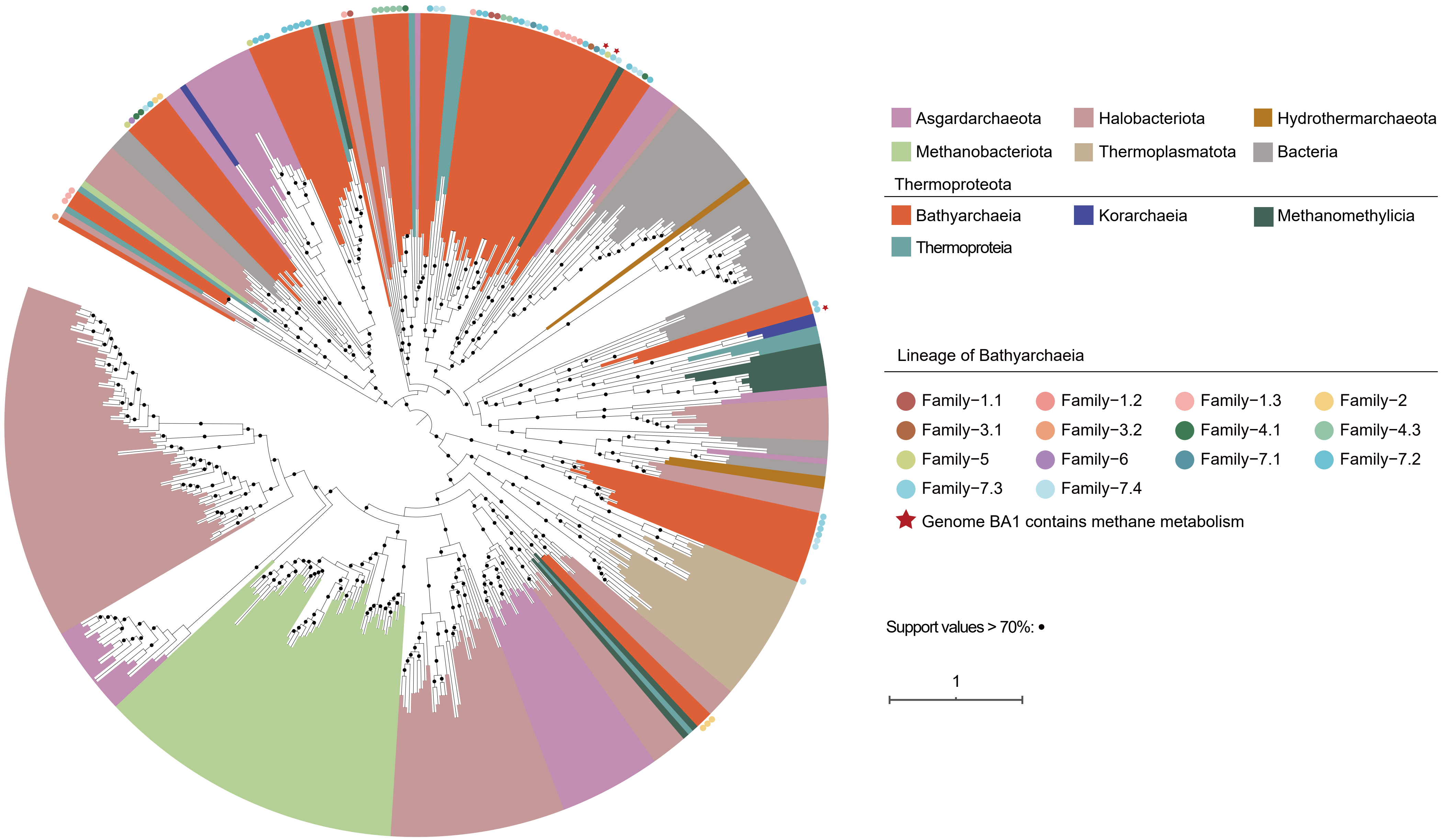

Asgardarchaeota
Halobacteriota
Hydrothermarchaeota
Methanobacteriota
Thermoplasmatota
Bacteria
Thermoproteota
Bathyarchaeia
Korarchaeia
Methanomethylicia
Thermoproteia
Lineage of Bathyarchaeia
Family−1.1
Family−1.2
Family−1.3
Family−2
Family−3.1
Family−3.2
Family−4.1
Family−4.3
Family−5
Family−6
Family−7.1
Family−7.2
Family−7.3
Family−7.4
Genome BA1 contains methane metabolism
Support values > 70%: •
1
